# Core–Shell Structured Metal–Organic Frameworks for pH-Triggered Combination Photodynamic/Chemotherapy-Based Cancer Treatment

**DOI:** 10.34133/bmr.0138

**Published:** 2025-01-22

**Authors:** Bei Liu, Huijuan Duan, Lirong Sun, Zechao Liu, Zhaogang Sun, Hongqian Chu

**Affiliations:** ^1^ College of Science, Minzu University of China, Beijing 100081, China.; ^2^ Translational Medicine Center, Beijing Chest Hospital, Capital Medical University/Beijing Tuberculosis and Thoracic Tumor Research Institute, Beijing 101149, China.

## Abstract

The use of hypoxia-activated prodrugs is a promising strategy to address the limitations of photodynamic therapy (PDT) caused by a hypoxic tumor microenvironment. However, the controlled release of these hypoxia-activated prodrugs during PDT remains a challenge. In this study, we present a metal–organic framework (MOF) with a core–shell structure that can achieve a high PDT efficacy and on-demand release of hypoxia-activated prodrugs (AQ4N) for hypoxic tumor therapy. The nanocomposites were created by assembling zeolitic imidazolate frameworks (ZIF-8) onto the surface of AQ4N-encapsulated porphyrinic MOF, followed by surface functionalization with folic acid-conjugated polyethylene glycol. AQ4N is entrapped in the mesopores of MOFs, and it shows acidic environment-triggered release due to the degradation of the ZIF-8. When exposed to laser, porphyrinic MOFs can produce reactive oxygen species for PDT. At the same time, PDT exacerbates hypoxia at the tumor site, leading to the bioreduction of AQ4N to AQ4 for enhanced anticancer activity. This work presents a practical approach to improve the tumor-targeting and therapeutic efficiency of hypoxic tumors.

## Introduction

Photodynamic therapy (PDT) has shown great potential for treating tumors with inherent noninvasive nature, high safety, high selectivity, and minimal systemic toxicity [1–3]. However, PDT’s therapeutic efficacy for killing hypoxic tumor cells is limited because of the requirement of O_2_ for producing reactive oxygen species (ROS) during PDT [4,5]. To overcome this, much effort has been devoted to developing the PDT system for hypoxic tumors, including oxygen-nanocarrier strategy [6,7], oxygen-generating strategy [8,9], and designing of oxygen-independent PDT [10–12]. One method that has attracted much attention is the use of hypoxia-activated prodrugs (e.g., tirapazamine, banoxantrone [AQ4N], and evofosfamide) during PDT [13–15]. These prodrugs can be reduced from nontoxic molecules to toxic drugs by tumor hypoxia, thus enabling a cascade therapy for hypoxic tumors. Despite extensive research and rapid growth, the controlled encapsulation and on-demand delivery of photosensitizers (PSs) with hypoxia-activated prodrugs still present big challenges.

Porphyrinic metal–organic frameworks (MOFs), such as PCN-224 or PCN-222, are promising nano-photosensitizers (nPSs) for PDT and have stimulated great research interests. They self-assemble from porphyrin PSs and metal ions/clusters through coordination interaction [16,17]. These MOF-based nPSs can keep the porphyrin PSs in their monomeric form to prevent self-quenching. Additionally, the porous structure of MOFs facilitates the diffusion of ROS and enables drugs encapsulated within nanopores to yield drug-delivery nPS [18–20]. In previous works, the porphyrinic MOFs have been used to encapsulate immunotherapy agent (e.g., indoleamine 2,3-dioxygenase) [18], tirapazamine [19], or doxorubicin [20] for combining chemotherapy or immunotherapy with PDT. However, the sustained drug release mechanism of these MOFs can result in high toxicity on the normal tissue/cells and insufficient drug concentration for tumor treatment.

One of the promising strategies to tackle this challenge is to develop stimuli-responsive drug delivery nanosystems, which can achieve on-demand drug release profiles triggered by some specific stimuli, such as pH, laser, or temperature [21]. In particular, studies on pH-responsive nanocarriers have received much attention in cancer therapy, because there is a notable difference between the normal tissue (pH ~7.0) and the tumor (pH ~5.5) [22–24]. Zeolitic imidazolate frameworks (ZIF-8), which is composed of Zn^2+^ ions and 2-methylimidazolate linkers, is a typical pH-responsive candidate [25]. Since the coordination bonds between Zn^2+^ ions and 2-methylimidazolate ligands can dissociate at pH 5.0 to 6.0 due to the protonation effect, ZIF-8 was degraded in the acidic tumor microenvironment (TME), while remaining stable under normal physiological conditions. Moreover, ZIF-8 is easy to fabricate, and the raw materials required for ZIF-8 synthesis are inexpensive. Though effective, the construction of a ZIF-8-based nanosystem combining PDT and hypoxia-activated prodrugs was still unsuccessful to date.

**Fig. 1.**
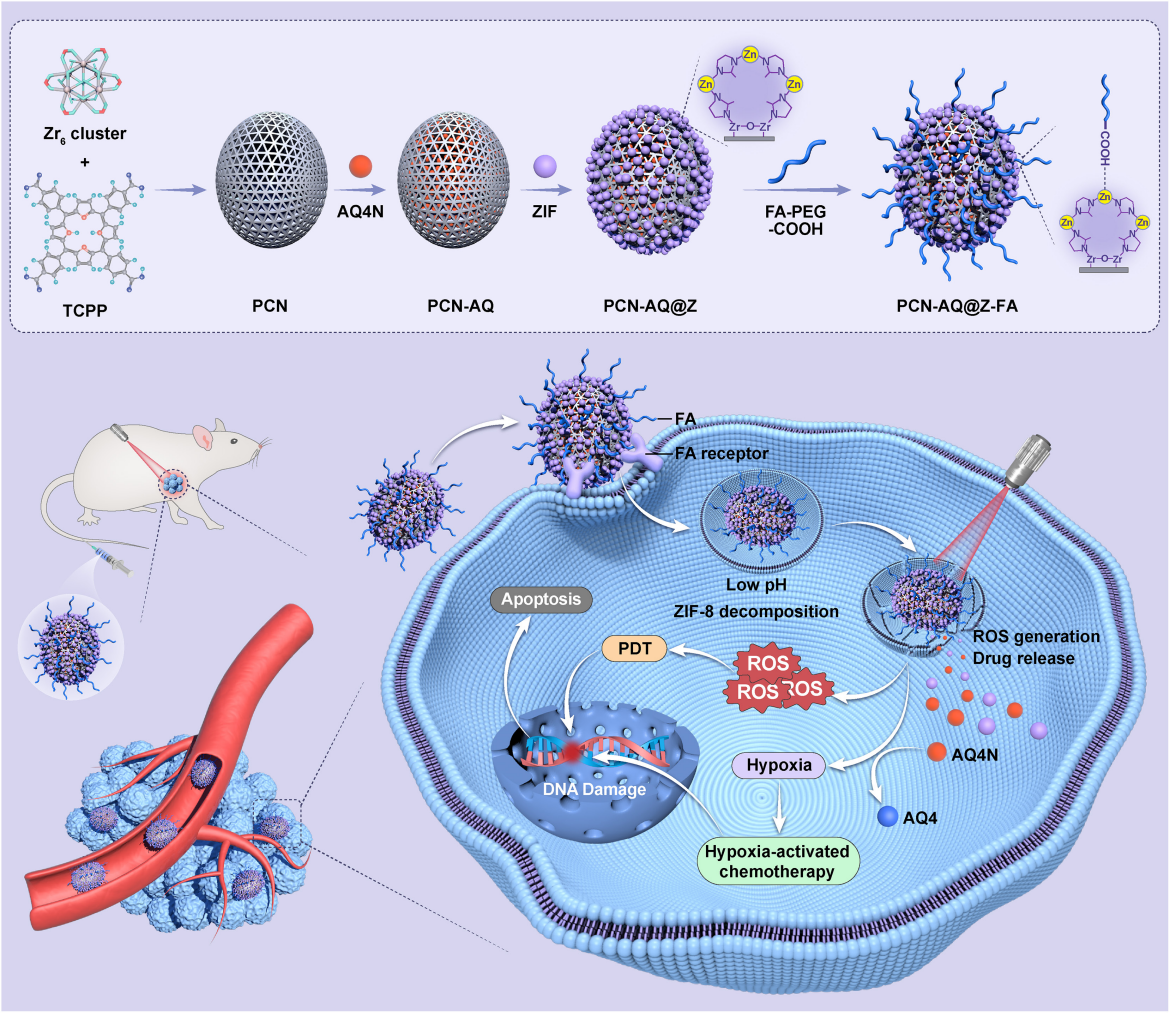
Schematic illustration of the synthesis of PCN-AQ@Z-FA and their bioapplication for tumor treatment through a combination of PDT and hypoxia-activated chemotherapy.

Herein, a ZIF-8-functionalized PCN nanoplatform has been successfully fabricated for the photodynamic/chemo-combinational therapy against hypoxic tumors. The mesoporous structure of PCN core not only allows an effective photo-induced generation of ROS for PDT, but also has a high drug-loading capacity of the hypoxia-activated prodrugs (AQ4N). The nontoxic ZIF-8 shell plays an important role of “gatekeepers” to block the AQ4N in the mesopores of PCN until it is dissociated in the acidic TME. Folic acid (FA)–polyethylene glycol (PEG) was also functionalized through coordination interactions to give the nanoplatform a specific biorecognition capability for targeting tumors. The resulting nanocomposites, labeled as PCN-AQ@Z-FA, exhibit unique antitumor properties, including enhanced active targeting effect toward cancer cells, an acidic environment-triggered drug release profile, effective singlet oxygen production for PDT, and efficient bioreduction of AQ4N to AQ4 at the tumor site (Fig. 1). Our results from both in vitro and in vivo studies demonstrated that these MOF-based nanoplatforms show great promise for pH-triggered combinational photodynamic/chemotherapy against hypoxic tumors.

## Materials and Methods

### Materials and chemicals

AQ4N, benzoic acid (99.5%), methanol (99.5%), N,N-dimethylformamide (DMF), and dimethyl sulfoxide (DMSO) were bought from the Aladdin Reagent Co. (Shanghai, China). 5,10,15,20-Tetrakis (4-carboxyphenyl) porphyrin (H_2_TCPP) and 1,3-diphenylisobenzofuran (DPBF) were purchased from TCI. Zinc nitrate hexahydrate, zirconyl chloride octahydrate, and 2-methylimidazole were purchased from Sigma-Aldrich. Annexin V-fluorescein isothiocyanate (FITC)/propidium iodide (PI) was purchased from Sangon Biotech Co., Ltd. (Shanghai, China). 2′,7′-Dichloroflorescein diacetate (DCFH-DA) and Hoechst 33342 were provided by Beyotime Biotechnology Co., Ltd. (Shanghai, China). Cell Counting Kit-8 (CCK-8) was bought from Dojindo (Japan Tongren). The ROS-ID Hypoxia/Oxidative Stress Detection Kit was bought from Enzo Life Sciences (New York, USA). Dulbecco’s modified eagle’s medium (DMEM), Roswell Park Memorial Institute (RPMI) 1640 medium, phosphate-buffered saline (PBS), fetal bovine serum (FBS), trypsin, and penicillin–streptomycin were obtained from Wisent Bio Co., Ltd. (Nanjing, China).

### Characterization

Transmission electron microscopy (TEM) and scanning electron microscopy (SEM) images were taken on a JEM-1400 transmission electron microscope and a JSM-7500F scanning electron microscope, respectively. UV–Vis–NIR (ultraviolet–visible–near infrared) absorption spectra were recorded on a JASCO V-550 spectrophotometer. In vitro cytotoxicity assay was obtained by a Thermo MULTISCAN GO reader (Thermo, USA). The flow cytometry assays were carried out using a Guava easyCyte flow cytometer 6 (Guava Technologies Inc., Hayward, CA, USA). Confocal laser scanning microscopy (CLSM) images were obtained using an Olympus FV1000 confocal microscope (Olympus Co., Japan). The in vivo fluorescence imaging was performed with an IVIS Spectrum in vivo imaging system (PerkinElmer Inc., Waltham, MA). The complete blood counts and the blood chemistry were tested by Celltac MEK-6318K (NIHON KOHDEN, Japan) and an automatic biochemical analyzer (Hitachi-7020, Japan), respectively.

### Synthesis of PCN-AQ@Z-FA

Two milliliters of DMF containing H_2_TCPP (2.6 μmol), zirconyl chloride octahydrate (18.7 μmol), benzoic acid (0.46 mmol), and water (20 μl) was added in a 5-ml flask. The mixture was heated at 90 °C. After stirring for 5 h, the PCN nanoparticles (NPs) were washed with DMF 2 times and finally redispersed in 2 ml of methanol. For the synthesis of PCN-AQ, 1 ml of PCN NPs was added into 2 ml of AQ4N solution (0.2 mg/ml, in methanol). After stirring at 37 °C for 12 h, AQ4N-loaded PCN (PCN-AQ) was centrifuged and redispersed in 1 ml of methanol. For the synthesis of PCN-AQ@Z, the PCN-AQ solution was mixed with zinc nitrate hexahydrate solution (2.975 mg/ml). After slowly stirring at 37 °C for 30 min, 2-methylimidazole methanol solution (4.926 mg/ml) was added to the mixed solution. The mixture was stirred for another 24 h. Then, the PCN-AQ@Z was collected by centrifugation. Finally, for the synthesis of PCN-AQ@Z-FA, the PCN-AQ@Z was mixed with 1 ml of PEG-FA-COOH solution (8 mg/ml). The uniformly dispersed suspension was stirred at 37 °C for 2 h and washed 3 times with fresh DMSO to remove the uncoordinated FA.

### Quantitative analysis of TCPP in NPs

For the quantitative analysis of TCPP in PCN or PCN-AQ@Z-FA NPs, a standard curve of TCPP was established. Briefly, the absorbance intensity of the TCPP solution (dissolved in sodium hydroxide solution with pH = 11) was measured using a UV–vis spectrophotometer (414 nm). Then, an absorbance intensity–TCPP concentration plot was built based on a series of TCPP concentrations, resulting in a fitted straight line *y* = 0.51839*x* + 0.02224, *R*^2^ = 0.998. After that, 40 μl of PCN was added into 3 ml of sodium hydroxide solution with pH = 11, followed by stirring at room temperature for 24 h. The absorbance of PCN solution at 414 nm was measured, and the concentration of TCPP in PCN could be calculated based on the standard curve of TCPP. The loading content (LC) of TCPP in PCN was calculated according to the following equation: LC (%) = [(weight of loaded TCPP)/(weight of PCN)] × 100%. The LC of TCPP in PCN-AQ@Z-FA was calculated according to the following equation: LC (%) = [(weight of loaded TCPP)/(weight of PCN-AQ@Z-FA)] × 100%.

### ROS detection

The singlet oxygen generation abilities of PCN-AQ@Z-FA were tested by utilizing DPBF as ^1^O_2_ chemical probe. Briefly, a certain amount of DPBF was dissolved in DMSO to form a DPBF/DMSO solution (1.5 mg ml^−1^). Then, the newly prepared DPBF solution (20 μl) was added into the PCN-AQ@Z-FA aqueous solution (3 ml). A 640-nm laser (100 mW cm^−2^) was used to irradiate the mixed solution for various time periods (0, 1, 2, 3, 4, 5, 6, 7, 8, 9, and 10 min), and the UV–Vis absorption spectra were recorded on a JASCO V-550 spectrophotometer. The absorption intensity of DPBF at 417 nm was used for comparison.

### The loading capacity and in vitro release of AQ4N

For the quantitative analysis of AQ4N in NPs, a standard curve of AQ4N was firstly established. Briefly, the absorbance intensity of the AQ4N aqueous solution was measured using a UV–Vis spectrophotometer. Then, an absorbance intensity-AQ4N concentration plot was built based on a series of AQ4N concentrations, resulting in a fitted straight line *y* = 0.03147*x* − 0.00169, *R*^2^ = 0.999. To calculate the drug-loading capacity of AQ4N in PCN-AQ@Z-FA, the supernatants were carefully collected. Based on the AQ4N’s standard curve, the AQ4N amount in the supernatant was calculated. The LC of AQ4N in PCN-AQ@Z-FA was calculated according to the following equation: LC (%) = [(the weight of AQ4N added − weight of AQ4N in supernatant)/(weight of PCN-AQ@Z-FA)] × 100%. For pH-responsive drug-releasing analysis, the pH-responsive release of AQ4N was investigated as follows: PCN-AQ@Z-FA NPs or PCN-AQ NPs were dispersed in 3 ml of aqueous solution with different pH values (pH = 7.0 or 5.5). After magnetic stirring for different times (e.g., 0, 1, 2, 4, 6, 12, and 24 h), the NPs were collected by centrifugation and the precipitates were redispersed in 3 ml of fresh media. The supernatant was carefully collected for the absorbance analysis. The release amount of AQ4N was determined according to the standard curve of AQ4N.

### Cellular uptake

MCF-7 cells and 4T1 cells were cultured in DMEM medium and RPMI 1640 medium containing 10% FBS and 1% antibiotics, respectively. Cellular uptake behavior was tested by CLSM and flow cytometry. Briefly, MCF-7 cells or 4T1 cells were seeded in a glass-bottomed confocal dish (for CLSM test) or a 6-well plate (for flow cytometry test) and cultured for 24 h. Subsequently, the cells were treated with PCN-AQ@Z and PCN-AQ@Z-FA (PCN equivalent 50 μg/ml) in Opti-MEM medium for 4 h. The cells were then washed with PBS and stained with Hoechst 33342. The CLSM images were obtained using an Olympus FV1000 confocal microscope. For flow cytometry test, the cancer cells were carefully collected and the intracellular fluorescence of AQ4N was analyzed.

### Cell studies

#### In vitro cytotoxicity test

To test the in vitro cytotoxicity of NPs, 16HBE cells (cultured in RPMI 1640 medium), MCF-7 cells, or 4T1 cells were seeded in 96-well plates at a density of 10^4^ cells/well and cultured for 24 h. Then, the cells were incubated with PCN@Z, PCN@Z-FA, PCN-AQ@Z, PCN-AQ@Z-FA (PCN equivalent 50 μg/ml), and Free-AQ4N in Opti-MEM medium. After 4 h of incubation, for the light irradiation groups, the cells were irradiated with a 640-nm laser for 10 min (100 mW cm^−2^). For the nonirradiation groups, the cells were cultured in the dark. After another 24 h, 10 μl of CCK-8 was added into each well. One hour later, the absorbance was measured by a microplate spectrophotometer at 450 nm.

#### Cell apoptosis analysis

Tumor cells were seeded in glass-bottomed confocal dishes (for CLSM test) or a 6-well plate (for flow cytometry test) and cultured for 24 h. Then, the cells were treated with PCN@Z, PCN@Z-FA, PCN-AQ@Z, PCN-AQ@Z-FA (PCN equivalent 50 μg/ml), and Free-AQ4N in Opti-MEM medium for 4 h. After being washed by PBS, the cells in irradiation groups were irradiated by a 640-nm laser (100 mW cm^−2^) for 10 min. For the nonirradiation groups, the cells were cultured in the dark. After incubation for another 1 h, the cells were incubated with calcein AM/PI for 20 min for live/dead cell staining. Finally, the cells were imaged by CLSM. For the flow cytometry test, the cancer cells were collected and the intracellular fluorescence of Annexin V-FITC/PI was analyzed.

#### Intracellular ROS/hypoxia detection

The intracellular ROS level was tested using DCFH-DA, a typical ROS fluorescent probe. Briefly, tumor cells were plated on glass-bottomed confocal dishes and cultured for 24 h. Then, the cells were treated with PCN@Z, PCN@Z-FA, PCN-AQ@Z, PCN-AQ@Z-FA (PCN equivalent 50 μg/ml), and Free-AQ4N in Opti-MEM medium for 4 h. After being washed with PBS, the cells in irradiation groups were irradiated by laser (640 nm, 100 mW cm^−2^) for 10 min. Then, the cells were incubated with DCFH-DA for 20 min, and the intracellular fluorescence was observed by CLSM.

For the intracellular ROS/hypoxia detection, MCF-7 cells were seeded in confocal dishes with a density of 2 × 10^5^ cells per well at 70% to 80% confluency. Then, the culture medium was removed, 100 μl of the ROS-ID Hypoxia/Oxidative Stress Detection Mix that contains PCN@Z or PCN@Z-FA NPs (PCN equivalent 50 μg/ml), ROS inducer control (pyocyanin), or hypoxia inducer control (DFO) was added. The positive control cells treated with ROS inducer or hypoxia inducer were included in experiments according to the manufacturer’s instructions. The experimental cells were incubated with PCN@Z and PCN@Z-FA for 4 h. Then, the cells were irradiated by laser (640 nm, 100 mW cm^−2^) for 10 min. After carefully removing the above reagent, the nuclei were stained by Hoechst 33342. Finally, the tumor cells were washed with PBS 3 times and imaged by CLSM.

#### Intracellular HIF-1α detection

For the intracellular HIF-1α detection, MCF-7 cells were plated on confocal dishes with a density of 2 × 10^5^ cells per well at 70% to 80% confluency. Then, the culture medium was removed and the cells were treated with PCN-AQ@Z-FA (PCN equivalent 50 μg/ml) and Free-AQ4N in Opti-MEM medium for 4 h. After being washed by PBS, the cells in light groups were irradiated by laser (640 nm, 100 mW cm^−2^) for 10 min. The intracellular HIF-1α detection was then detected as follows: Cells were firstly fixed with 4% paraformaldehyde in PBS, pH 7.4, for 10 min, and then permeabilized with 0.2% Triton X-100 at 37 °C for 10 min. The cells were washed in PBS 3 times for 5 min, and then incubated with 1% bovine serum albumin (BSA) for 30 min to block unspecific binding of the antibodies. After that, the cells were stained with HIF-1α antibody overnight at 37 °C. The cells were washed 3 times with PBS for 5 min and then incubated with the secondary antibody (Goat Anti-Rabbit IgG H&L [Alexa Fluor 488] 1:500) in 1% BSA for 1 h at room temperature in the dark. The cells were washed with PBS 3 times. Finally, the cells were stained with Hoechst 33342 for CLSM observation.

### Animal experiments

#### In vivo bioimaging

Female BALB/c mice aged 6 to 8 weeks with a body weight of 18 to 20 g were purchased from Beijing Vital River Laboratory Animal Technology Co., Ltd. All in vivo experiments were carried out complying with NIH guidelines, and the experimental protocol was approved by the Institutional Animal Care and Use Committee of Beijing Tuberculosis and Thoracic Tumor Research Institute. 4T1 tumor-bearing mice models were established by injecting 1 × 10^6^ 4T1 cancer cells into the specific part of the BALB/c mice. For in vivo bioimaging, mice were treated with PCN-Cy5@Z or PCN-Cy5@Z-FA NPs, and the whole-body fluorescence imaging of tumor-bearing mice was collected at specified time intervals (1, 3, 6, 12, 24, and 48 h) after intravenous administration. Also, the fluorescence imaging of the main organs (tumor, heart, liver, spleen, lung, and kidney) from the tumor-bearing mice after intravenous administration of PCN-Cy5@Z-FA was collected at specified time intervals.

#### In vivo antitumor therapy

4T1 tumor-bearing mice models were established by injecting 4T1 cells (1 × 10^6^ cells with 100 μl, 1:1 [v/v] PBS, and Matrigel) into the right back of the BALB/c mice. After 1, 3, and 5 days, the mice were intravenously injected with PCN@Z, PCN@Z-FA, PCN-AQ@Z, PCN-AQ@Z-FA, and Free-AQ4N (100 μl, PCN dose: 20 mg/kg). At 6 h after injection, the mice in irradiation groups were exposed to laser (640 nm, 100 mW cm^−2^) for 10 min. The changes of primary tumors and the body weights of the mice were monitored every 2 days. On the 16th day after the first administration, the mice were sacrificed and major tissues (tumor, heart, liver, spleen, lung, and kidney) were fixed with 4% paraformaldehyde and then taken to perform histological assay. The mice blood was collected from retroorbital veins and centrifuged to obtain serum. The complete blood counts were analyzed and the blood chemistry was tested using an automatic biochemical analyzer.

## Results

### Synthesis and characterization of PCN-AQ@Z-FA

**Fig. 2.**
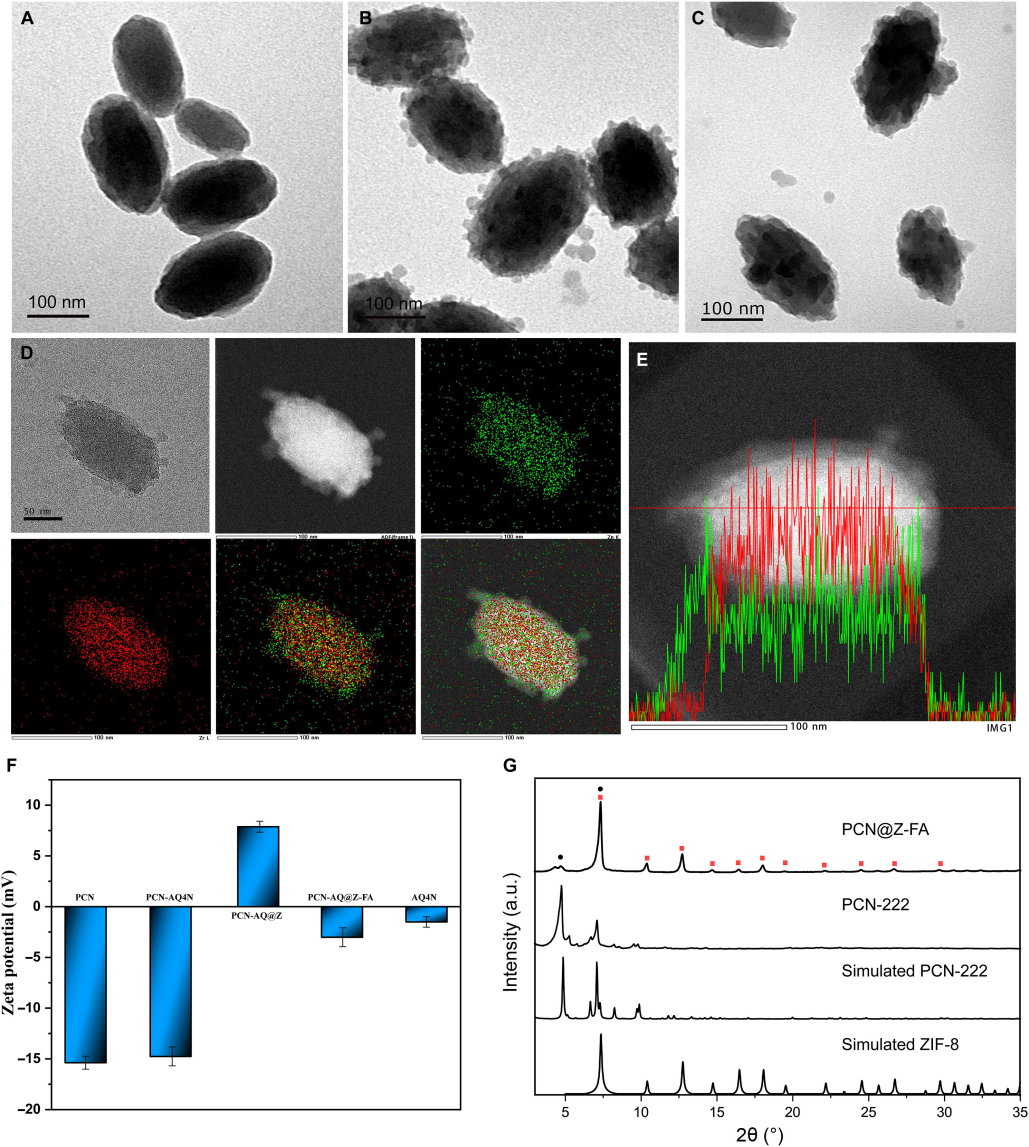
Characterization of PCN, PCN-AQ@ZIF, and PCN-AQ@Z-FA. TEM images showing the morphology of (A) PCN, (B) PCN-AQ@Z, and (C) PCN-AQ@Z-FA. Scale bar is 100 nm. (D and E) Elemental mapping images and energy-dispersive x-ray spectroscopy of PCN-AQ@Z-FA (red represents Zr and green represents Zn). (F) Zeta potentials of PCN, PCN-AQ, PCN-AQ@Z, PCN-AQ@Z-FA, and Free-AQ4N. (G) Powder x-ray diffraction (PXRD) patterns of PCN-222 and PCN@Z-FA.

Figure 1 presented a diagram of the synthesis process of PCN-AQ@Z-FA and their application for tumor treatment. Firstly, nanoscale PCN was synthesized using a previously established technique [26,27]. The PCN NPs showed a typical shuttle-like shape with an average diameter of 145 nm as shown by SEM (Fig. S1) and TEM (Fig. 2A) images. Benefiting from their high porosity and large surface area, PCN NPs are selected as appealing nanocarriers for drug payloads. AQ4N, a kind of hypoxia-activated antitumor prodrugs, was encapsulated in the porous structure of PCN through simple impregnation mechanism [28]. TEM of PCN-AQ showed a similar size and morphology with PCN (Fig. S2), indicating that AQ4N encapsulated in the nanopores of PCN cannot affect the particle size or morphology of NPs. Then, small ZIF-8 NPs were decorated on the surface of AQ4N-encapsulated PCN to block AQ4N molecules in the mesopores of PCN. As shown in Fig. 2B and Fig. S3, small ZIF-8 NPs can be clearly observed on the surface of PCN. To give PCN-AQ@Z a specific biorecognition capability and prolonged blood circulation time, FA-PEG was conjugated on PCN-AQ@Z through a coordination interaction between the carboxy groups of FA-PEG with coordinatively unsaturated Zn^2+^ sites of ZIF-8 [29,30]. The resulting PCN-AQ@Z-FA NPs remained monodispersed in size without obvious shape change and aggregation, as shown in Fig. 2C. The elemental mapping (Fig. 2D) and line scan (Fig. 2E) provided further evidence for the formation of PCN-AQ@Z-FA. Both elemental mapping study and EDS line scan analysis of Zr and Zn on a single PCN-AQ@Z-FA NP confirmed their core–shell structure in terms of geometrical and compositional distributions. The level of the Zr element remained steady in the core domain, while the counts for the Zn element increased at the location of the shell part.

Dynamic light scattering measurements were performed to determine the hydrodynamic size of PCN, PCN-AQ@Z, and PCN-AQ@Z-FA. The findings showed that the size of PCN-AQ@Z-FA NPs increased from 145 to 190 nm after coating with ZIF and modification with FA-PEG (Fig. S4). Zeta potential measurements were performed to determine the surface potentials of PCN, PCN-AQ, PCN-AQ@Z, and PCN-AQ@Z-FA. Figure 2F indicates that PCN had a negative charge (−15.4 mV). After the surface coating of ZIF-8, the zeta potential shifted to a positive value (+7.8 mV) because of the Zn^2+^-rich surface of the nanoscale ZIF-8 shell. The modification of FA-PEG further reduced the zeta potential to –3.0 mV due to the existence of carboxyl negative groups in FA-PEG. Note that there was no obvious difference in zeta potentials of PCN before and after AQ4N loading, indicating that AQ4N encapsulated in the nanopores of PCN did not affect the zeta potential of NPs. Additionally, the UV–Vis–NIR absorption spectra of PCN-AQ@Z-FA displayed typical absorption peaks of porphyrinic MOFs, and the absorption intensity steadily increased with the rising concentrations of PCN-AQ@Z-FA (Fig. S5).

X-ray diffraction (XRD) was conducted to identify the crystal structure and phase composition of samples. As shown in Fig. 2G, the XRD pattern of the as-synthesized PCN power presented a perfect match with the standard pattern of PCN-222 MOFs. The XRD pattern of PCN@Z-FA showed some diffraction peaks from PCN-222 besides ZIF-8, indicating the successful fabrication of the core–shell structured PCN@Z NPs. No diffraction peaks of FA were detected in the XRD of PCN@Z-FA, suggesting that the FA modification in PCN@Z-FA had less effect on the XRD pattern of PCN@Z. Brunauer–Emmett–Teller (BET) isotherms of NPs were also collected to analyze their surface area, pore size, and pore distribution. As presented in Fig. S6A, PCN, PCN-AQ, and PCN-AQ@Z showed typical characteristics of type IV isotherms, indicating that similar mesoporous structures exist in these NPs. The results were also proved by their similar pore size distributions of PCN, PCN-AQ, and PCN-AQ@Z (Fig. S6B). Notably, a relatively low BET surface area (213.5 m^2^/g) and total pore volume (0.29 cm^3^/g) of PCN-AQ were detected when compared with PCN (287.3 m^2^/g and 0.32 cm^3^/g), suggesting that a certain degree of pores in PCN-AQ were filled with AQ4N molecules. Further decoration of ZIF-8 onto the surface of PCN-AQ increased the BET surface area to 285.9 m^2^/g and the total pore volume to 0.43 cm^3^/g, perhaps because of the rough surface of PCN-AQ@Z and the intrinsic porosity of the ZIF shell.

### Release of AQ4N and TCPP-mediated ^1^O_2_ generation

**Fig. 3.**
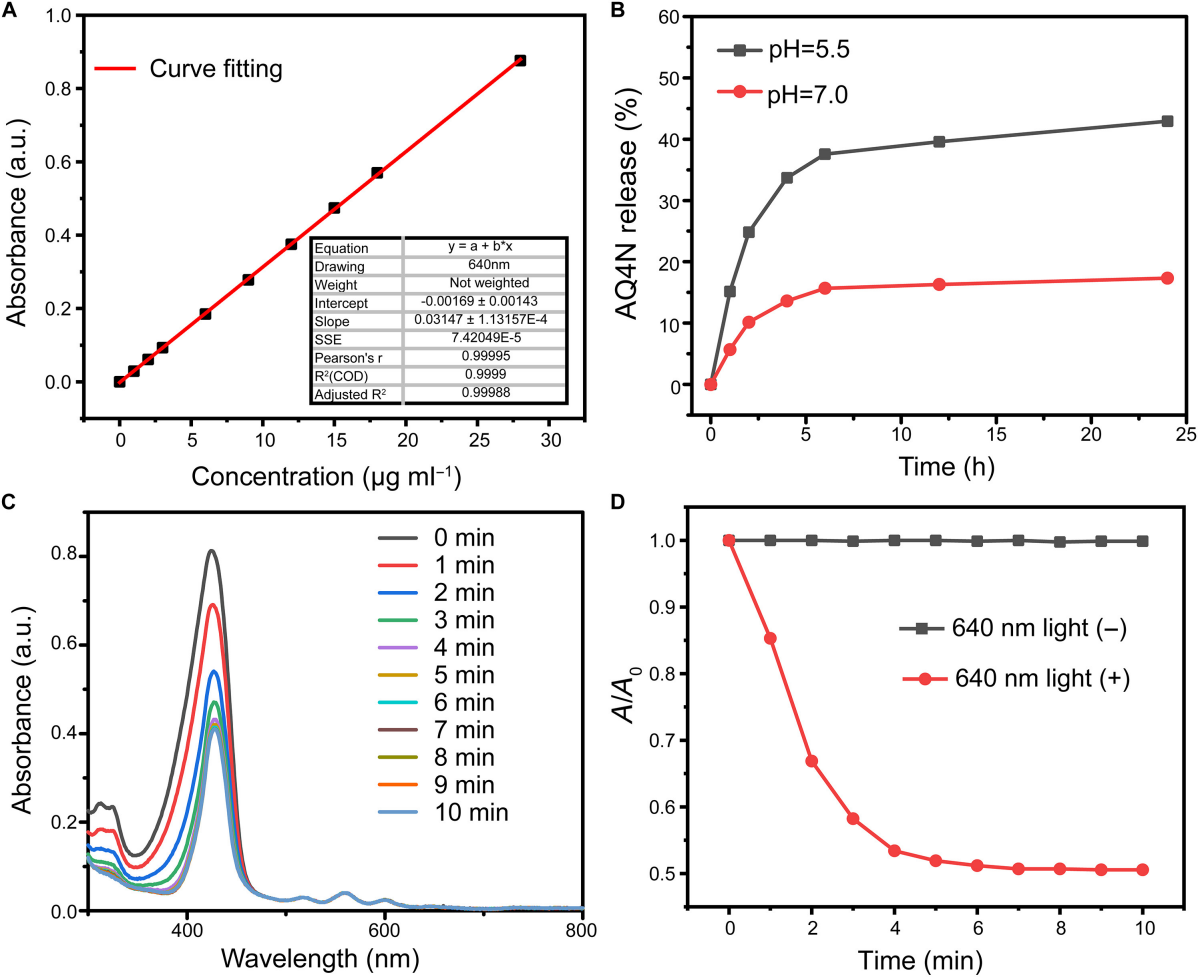
Release of AQ4N and TCPP-mediated ^1^O_2_ generation. (A) The standard curve of AQ4N. (B) Drug release curves of PCN-AQ@Z-FA at room temperature, measured at various pH levels. (C) UV–Vis absorption spectra of PCN-AQ@Z-FA observed at different exposure times. (D) The fluorescence intensity of DPBF changes with time after treatment with PCN-AQ@Z-FA with or without 640-nm laser irradiation.

ZIF-8, a well-known pH-responsive MOF, was chosen as the protective shell of AQ4N-encapsulated PCN. As the coordination bonds of ZIF-8 could dissociate at acidic environment (pH 5.0 to 6.0) while remaining stable under normal physiological conditions (pH 7.0), we next explored the pH-responsive release profiles of AQ4N from PCN-AQ@Z-FA NPs. Firstly, a standard curve of AQ4N was tested. The curve was based on the linear relationship between different concentrations of AQ4N and their corresponding absorption intensities at 610 nm (Fig. 3A). Based on the standard curve of AQ4N, the AQ4N loading efficiency and capacity of PCN-AQ@Z-FA were calculated to be 58.9% and 8.2%, respectively. Then, AQ4N release profiles from PCN-AQ@Z-FA NPs were tested at 37 °C in aqueous solution with pH levels of 7.0 and 5.5 (Fig. 3B). It was found that the release of AQ4N was dependent on pH levels: the cumulative amount of released AQ4N reaching 44.3% at pH 5.5, which is much higher than the 15.2% at pH 7.0. The AQ4N release profiles from PCN-AQ were also tested. As presented in Fig. S7, a similar cumulative release amount of AQ4N was achieved at pH 7.0 (42.3%) compared to pH 5.5 (48.7%), mainly because there is no gatekeeper of ZIF-8 to block the AQ4N in the mesopores of PCN. Considering the mildly acidic environment of TME, the acidic environment-triggered release of AQ4N at the tumor site would cause much more damage to tumor cells while causing less destruction to normal cells.

The incorporation of TCPP as functional linkers presented opportunities to enable effective ROS generation of PCN. Firstly, the quantitative analysis of TCPP included in the NPs was conducted. An absorbance–concentration plot was built based on a series of TCPP concentrations, resulting in a fitted straight line *y* = 0.51839*x* + 0.02224, *R*^2^ = 0.998 (Fig. S8). According to this linear relationship, the LC of TCPP in PCN was calculated to be 34.9%, and the LC of TCPP in PCN@Z-FA was calculated to be 26.7%. ^1^O_2_ generation induced by PCN@Z-FA NPs under laser irradiation was then investigated using DPBF. DPBF is a specific scavenger for ^1^O_2_ that reacts with ^1^O_2_ via a 1,3-addition mechanism. The UV–Vis spectra of DPBF in NP solution were observed at different exposure times (0 to 10 min). Figure 3C and D shows that the absorption intensity of DPBF decreased in a time-dependent manner upon light irradiation. In contrast, the absorption remained unchanged without laser irradiation. These results demonstrated that the light photons absorbed by PCN@Z-FA NPs can efficiently transfer ^3^O_2_ to generate ^1^O_2_. In addition, the UV–Vis spectra of DPBF in Free-TCPP solution have also been investigated for comparison. The results exhibited that Free-TCPP had a similar ROS production with PCN@Z-FA NPs (Fig. S9), indicating that the PDT of PCN@Z-FA NPs was mediated by TCPP.

### Cellular internalization of PCN-AQ@Z-FA

**Fig. 4.**
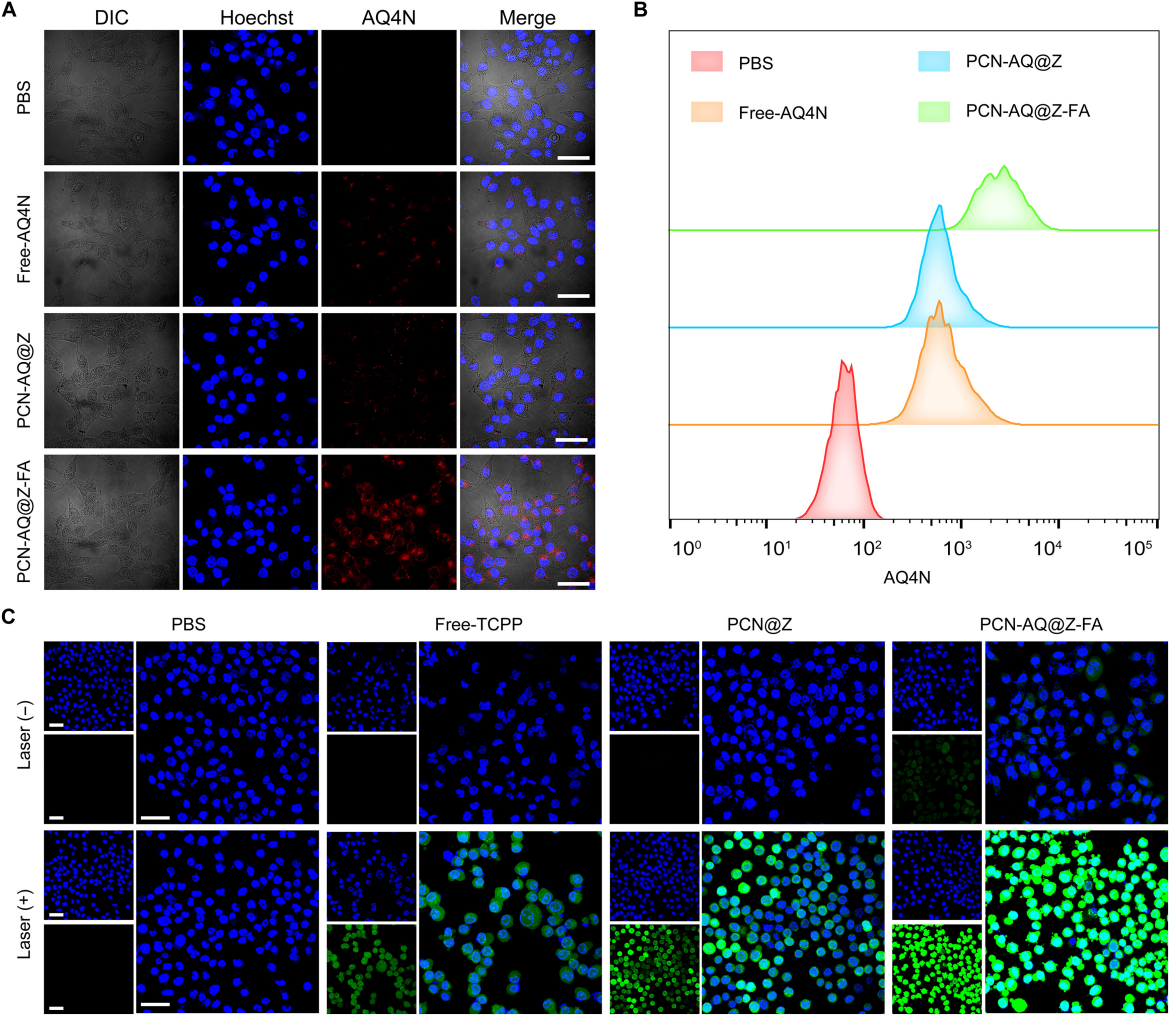
In vitro evaluation of cellular uptake and ROS production. (A) CLSM images of MCF-7 cells treated with PBS, Free-AQ4N, PCN-AQ@Z, and PCN-AQ@Z-FA at 37 °C for 4 h. MCF-7 were stained with Hoechst (blue). Scale bar, 50 μm. (B) Flow cytometry analysis of MCF-7 cells treated with PBS, Free-AQ4N, PCN-AQ@Z, and PCN-AQ@Z-FA. (C) CLSM analysis of ROS generation in MCF-7 cells treated with PBS, Free-TCPP, PCN@Z, and PCN@Z-FA. Scare bar, 50 μm.

Next, we explored the potential use of NPs for combinational photodynamic and chemotherapy. Firstly, the cellular internalization abilities of Free-AQ4N, PCN-AQ@Z, and PCN-AQ@Z-FA were analyzed by incubating these NPs or drugs with MCF-7 cells at 37 °C for 4 h. The results showed that the red fluorescence of AQ4N (excited by 633 nm laser) can be observed in the groups of PCN-AQ@Z or Free-AQ4N (Fig. 4A). Notably, PCN-AQ@Z-FA-treated cells showed a much higher fluorescence intensity than that treated with PCN-AQ@Z or Free-AQ4N, demonstrating the satisfactory targeting ability of PCN-AQ@Z-FA via FA-receptor-mediated endocytosis [31]. The flow cytometry analysis also supported this observation, which showed a 3.65-fold higher fluorescence intensity of MCF-7 cells treated with PCN-AQ@Z-FA than that of cells treated with PCN-AQ@Z (Fig. 4B and Fig. S10). Similar cellular uptake results were also observed by choosing 4T1 cells (with overexpressed FA receptors in the cell membrane) as the tumor cell model (Fig. S11). Control experiment was carried out on A549 cancer cells, which have a low level of FA-receptor expression. Figure S12 shows a similar low intracellular uptake efficiency when treated with PCN-AQ@Z and PCN-AQ@Z-FA.

To monitor the PCN-AQ@Z-FA NPs at the subcellular level following their endocytosis, the lysosomal compartments of MCF-7 cells were stained with LysoTracker Green. As shown in Fig. S13, a time-dependent enhancement of AQ4N fluorescence was clearly obtained, indicating the efficient internalization of PCN-AQ@Z-FA. Additionally, the red signal of AQ4N overlays with the green signal of LysoTracker, suggesting that the majority of PCN-AQ@Z-FA NPs accumulated inside endo/lysosomes along with the incubation time extension. When the cells were treated with PCN-AQ@Z-FA NPs followed by laser irradiation for 10 min, the red signals of AQ4N detached themselves from the lysosomal compartments (Fig. S14). The efficient lysosomal escape capability of PCN-AQ@Z-FA (+) was mainly attributed to the generation of cytotoxic ROS from PCN-AQ@Z-FA NPs, which can disrupt the lysosomal membranes through photochemical internalization, thus leading to the release of AQ4N for effective chemotherapy [32].

### Intracellular evaluation of ^1^O_2_ generation and AQ4N activation

Subsequently, we investigated the generation of ^1^O_2_ in MCF-7 cells using a widely used intracellular ^1^O_2_ indicator called DCFH-DA [33,34]. As shown in Fig. 4C, cells incubated with Free-TCPP, PCN@Z, and PCN@Z-FA without 640-nm laser irradiation showed little fluorescence signal of DCF. In contrast, after the 640-nm laser irradiation, MCF-7 cells showed a much higher fluorescence intensity of DCF, indicating the high generation amount of ROS in cells for the laser irradiation groups. The relative fluorescence intensity in the Free-TCPP (+) group was similar to that in the PCN@Z (+) group, which was consistent with the results of the DPBF assay. Notably, cells incubated with PCN@Z-FA following 640-nm laser irradiation exhibited a higher fluorescence intensity of DCF than that treated with PCN@Z (+) and Free-TCPP (+) groups due to the improved cell uptake of PCN@Z-FA NPs. 4T1 cells were also used to evaluate the intracellular ROS generation. As shown in Fig. S15, PCN@Z-FA (+)-treated 4T1 cells displayed brighter fluorescence of DCF, similar to the results in MCF-7 cells.

**Fig. 5.**
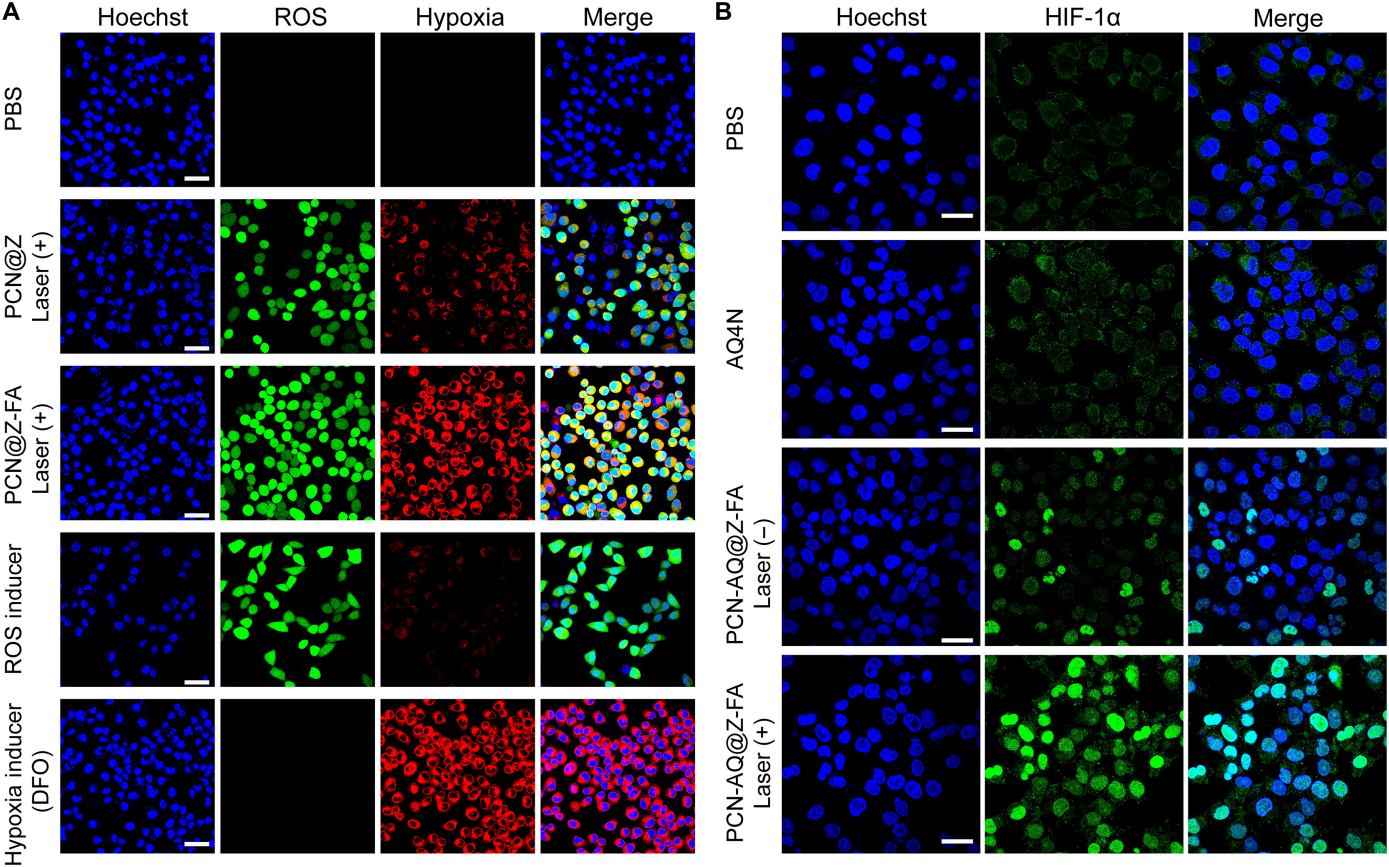
The evaluation of cellular hypoxia treated with the nanosystem and laser irradiation. (A) CLSM images of MCF-7 cells treated with PCN@Z (+), PCN@Z-FA (+), ROS inducer, and DFO (Deferoxamine) (blue represents Hoechst, green represents ROS, and red represents hypoxia). (B) The expression of HIF-1α in different groups was analyzed by immunofluorescence. Scare bar, 50 μm.

After analyzing the production of ROS in MCF-7 cells treated with PCN-AQ@Z-FA (+), we conducted further characterization of the hypoxia microenvironment induced by PDT treatment. To detect the hypoxia/ROS generation of MCF-7 cells after PCN@Z (+) or PCN@Z-FA (+) treatment, we used a hypoxia/oxidative stress detection kit [35,36]. MCF-7 cells treated with PBS were used as the negative control, and MCF-7 cells treated with ROS inducer (Pyocyanin, Pyo) or hypoxia inducer (Deferoxamine, DFO) were used as positive controls. As shown in Fig. 5A, the cells treated with PCN@Z (+) or PCN@Z-FA (+) exhibited green and red fluorescence signals, respectively. This was due to effective ROS generation and the subsequent hypoxia production. We noticed that PCN@Z-FA (+) showed a stronger fluorescence signal than that of PCN@Z (+) because of its active targeting of MCF-7 cells via FA-receptor-mediated endocytosis. To detect hypoxia in the cellular environment, we used hypoxia-inducible factor-1α (HIF-1α), a hypoxia-inducible heterodimeric transcription factor, as detection probes [37,38]. As shown in Fig. 5B, the cells treated with PCN-AQ@Z-FA (+) exhibited a much stronger intensity of green fluorescence, indicating the up-regulated expression of HIF-1α induced by PDT. These results showed a high level of light-induced hypoxia after PDT treatment, which was expected to enhance the anticancer activity of AQ4N.

As known, AQ4N can be reduced via an enzymatic reaction to cytotoxic AQ4 (a DNA affinity inserter) under hypoxic conditions [15]. Therefore, we next investigated the hypoxia activation of AQ4N to AQ4 in tumor cells. The intracellular behaviors of PCN-AQ@Z-FA and Free-AQ4N under normoxic or hypoxic conditions were imaged by CLSM (Fig. S16). As expected, in either the Free-AQ4N or PCN-AQ@Z-FA group, the red AQ4N fluorescence mainly concentrated in the cytoplasm region of cells under normoxic conditions, indicating that less AQ4N was converted into AQ4 (strong affinity with nuclear DNA). In contrast, the red fluorescent signals mainly accumulated in the nucleus under hypoxic conditions, suggesting that AQ4N has been reduced to hydrophobic AQ4 under a hypoxic environment. To sum up, AQ4N released from PCN-AQ@Z-FA can be reduced to AQ4 under a hypoxic environment, which can easily cross the nuclear membrane of tumor cells and bind to the nuclear DNA for tumor therapy.

### In vitro cell viability test

**Fig. 6.**
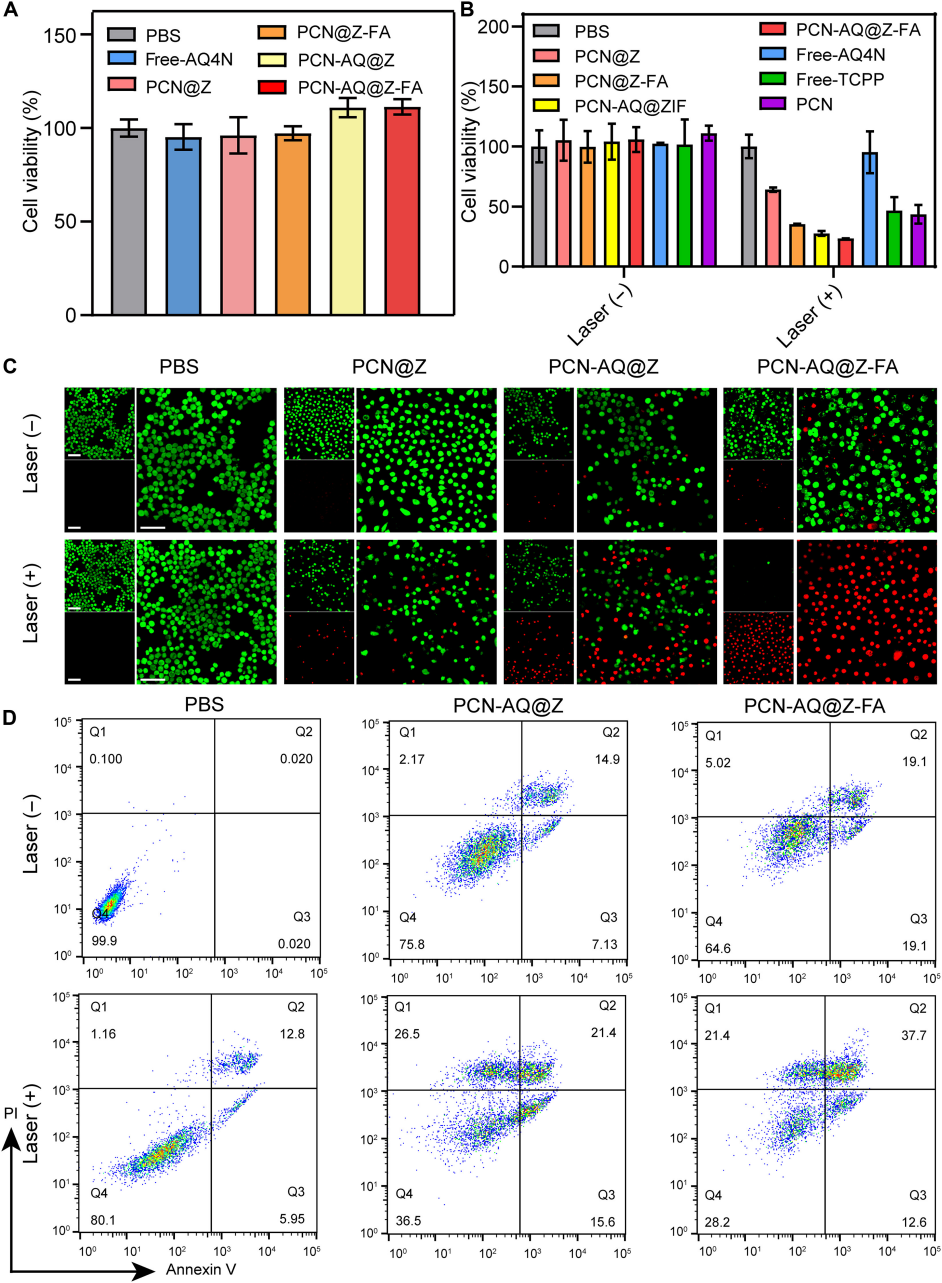
In vitro killing effects of the nanosystem. (A and B) CCK-8 assay was performed to evaluate (A) 16HBE and (B) MCF-7 cell viability upon different treatments. The data are presented as the mean ± SD (*n* = 5). (C and D) CLSM images (green represents live cells and red represents dead cells) and flow cytometry analysis of MCF-7 cells treated with PCN@Z, PCN-AQ@Z, and PCN-AQ@Z-FA. Scale bar, 50 μm.

A typical CCK-8 assay was conducted to test the cytotoxicity of NPs on normal cells (16HBE cells). The results in Fig. 6A showed that treatment with NPs had no marked effect on cell proliferation, indicating their low toxicity to 16HBE cells. To determine the dosage concentrations utilized for tumor-cell cytotoxicity experiments, the cytotoxicity profiles of PCN-AQ@Z-FA (PCN equivalent 0, 10, 30, 50, and 100 μg/ml) against MCF-7 cells were evaluated using CCK-8 assay. As presented in Fig. S17, cell viability treated with PCN-AQ@Z-FA (+) was decreased by increasing the concentrations of NPs from 0 to 50 μg/ml. However, when the concentration increased to 100 μg/ml, the photo-irradiated PCN-AQ@Z-FA group showed a similar cell viability to that of cells treated with 50 μg/ml of PCN-AQ@Z-FA. Therefore, we used 50 μg/ml for our following cell experiments. Moreover, to find out the effect of laser irradiation time on the cell viabilities, the relative viabilities of the cells after different laser irradiation times (1, 4, 7, and 10 min) were evaluated. As presented in Fig. S18, the cell viability decreased along with laser irradiation time and reached a stationary phase after 10 min of irradiation. Therefore, we chose 10 min as irradiation time in the following experiments. Next, the hypoxia-activated cytotoxicity of PCN-AQ@Z-FA NPs against MCF-7 cells was tested. Hypoxic cell culture environment with a low partial pressure of oxygen (2%) was conducted to simulate the hypoxic tumor condition. As presented in Fig. S19, treatment with PCN-AQ@Z-FA at different concentrations (of up to 100 μg/ml) resulted in no obvious change in the cell viability under normoxic conditions (oxygen levels of 21%), suggesting the good biocompatibility of the PCN-AQ@Z-FA NPs during the drug delivery process. In contrast, PCN-AQ@Z-FA exhibited significant cytotoxicity under hypoxia conditions (oxygen levels of 2%), suggesting the hypoxia-selective cytotoxicity of PCN-AQ@Z-FA NPs.

Then, the cytotoxicity of different NPs (PCN equivalent 50 μg/ml) against MCF-7 cancer cells was evaluated. For laser irradiation groups, we used the same conditions of the laser power density and irradiation time (100 mW cm^−2^, 10 min). As shown in Fig. 6B, PBS, Free-AQ4N, PCN@Z, PCN@Z-FA, PCN-AQ@Z, and PCN-AQ@Z-FA had no marked effect on cell viability, demonstrating the negligible toxicity of these NPs to cells. Treatment with PBS or Free-AQ4N following NIR irradiation did not result in a significant decrease in the cell viability, implying the low toxicity of laser irradiation to MCF-7 cells. In contrast, treatment with PCN@Z (+), PCN@Z-FA (+), PCN-AQ@Z (+), and PCN-AQ@Z-FA (+) markedly affected the cell viability. Notably, the cell inhibitory rates in the PCN-AQ@Z group (27.64%) and PCN-AQ@Z-FA group (23.32%) were higher than that in the PCN@Z (64.09%) group and PCN@Z-FA group (35.24%), indicating that an enhanced potency contributed to the tumor-targeting effect of FA. The laser-irradiated PCN-AQ@Z-FA group showed the lowest cell viability (23.32%) compared to the cells treated with PCN@Z-FA (+) (35.24%), suggesting a better therapeutic effect of combinational photodynamic/chemotherapy. Similar antitumor results can also be obtained by using 4T1 cells as the antitumor model. As presented in Fig. S20, the treatment with PCN-AQ@Z-FA (+) to 4T1 cells resulted in the highest cytotoxicity (83.1%) compared to the other groups, confirming the enhanced cell killing effect of PCN-AQ@Z-FA NPs through the tumor-targeted photodynamic/chemo-combined therapy.

The high cytotoxicity of PCN-AQ@Z-FA was further tested using the calcein AM/propidium iodide (PI) assay. The results indicated that MCF-7 cells treated with PCN-AQ@Z-FA (+) showed a significant fluorescence change from green to red, indicating the efficient killing effect of PCN-AQ@Z-FA on cancer cells (Fig. 6C and Fig. S21). The Annexin V-FITC/PI apoptosis detection assay also confirmed that PCN-AQ@Z-FA following laser irradiation caused the lowest level of healthy cells (28.2%) among all groups (Fig. 6D and Fig. S22).

### Targeting ability of PCN-AQ@Z-FA in vivo

**Fig. 7.**
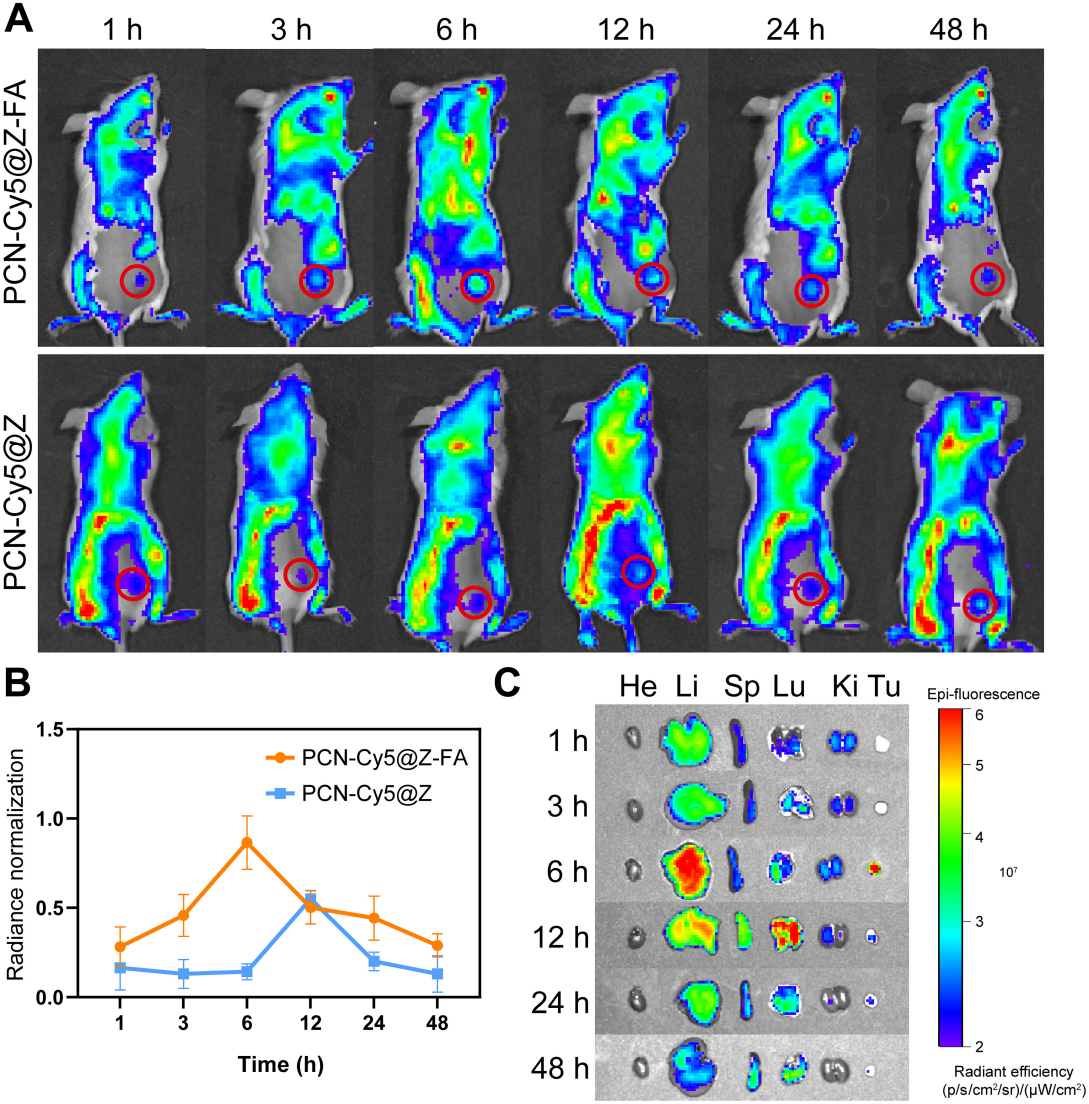
In vivo fluorescence imaging of the nanosystem. (A) Whole-body fluorescence imaging of tumor-bearing mice at specified time intervals (1 h, 3 h, 6 h, 12 h, 24 h, and 48 h) after intravenous administration of PCN-Cy5@Z-FA and PCN-Cy5@Z. Red circles indicate tumors. (B) Quantification of the fluorescence intensity at the tumor sites. Data are presented as mean ± SD (*n* = 5). (C) Ex vivo imaging of the tumor (Tu = tumor) and major organs (He = heart, Li = liver, Sp = spleen, Lu = lung, and Ki = kidney) upon PCN-Cy5@Z-FA injection at specified time intervals.

After observing the positive targeting ability of PCN-AQ@Z-FA in vitro, we further investigated its accumulation in vivo. We injected PCN-Cy5@Z-FA (where Cy5 was entrapped in the mesopores of PCN) in tumor-bearing mice intravenously. The results (Fig. 7A and B) showed that the fluorescence intensity of PCN-Cy5@Z-FA reached its maximum after 6 h of administration, which was approximately 1.39-fold higher than that at 1 h. The fluorescence signal remained up to 48 h, indicating efficient delivery of NPs to tumors. Note that the Cy5 signal from MOFs was detected across a broader area of mice in Fig. 7A, primarily due to their retention in pulmonary capillaries, the phagocytosis by the reticuloendothelial system in the liver and spleen, and the rapid metabolism by the kidneys. Similar results were reported for in vivo bioimaging evaluation of NPs [39,40]. Ex vivo imaging of main organs and tumors (Fig. 7C) showed that the maximal fluorescence intensity of the tumor was observed at 6 h after administration, which is consistent with the previous results. Notably, mice treated with PCN-Cy5@Z-FA showed a much stronger fluorescence intensity at the tumor site than those treated with PCN-Cy5@Z, indicating the efficient tumor-targeting ability of NPs (Fig. 7A and B).

### In vivo antitumor effect of PCN-AQ@Z-FA

**Fig. 8.**
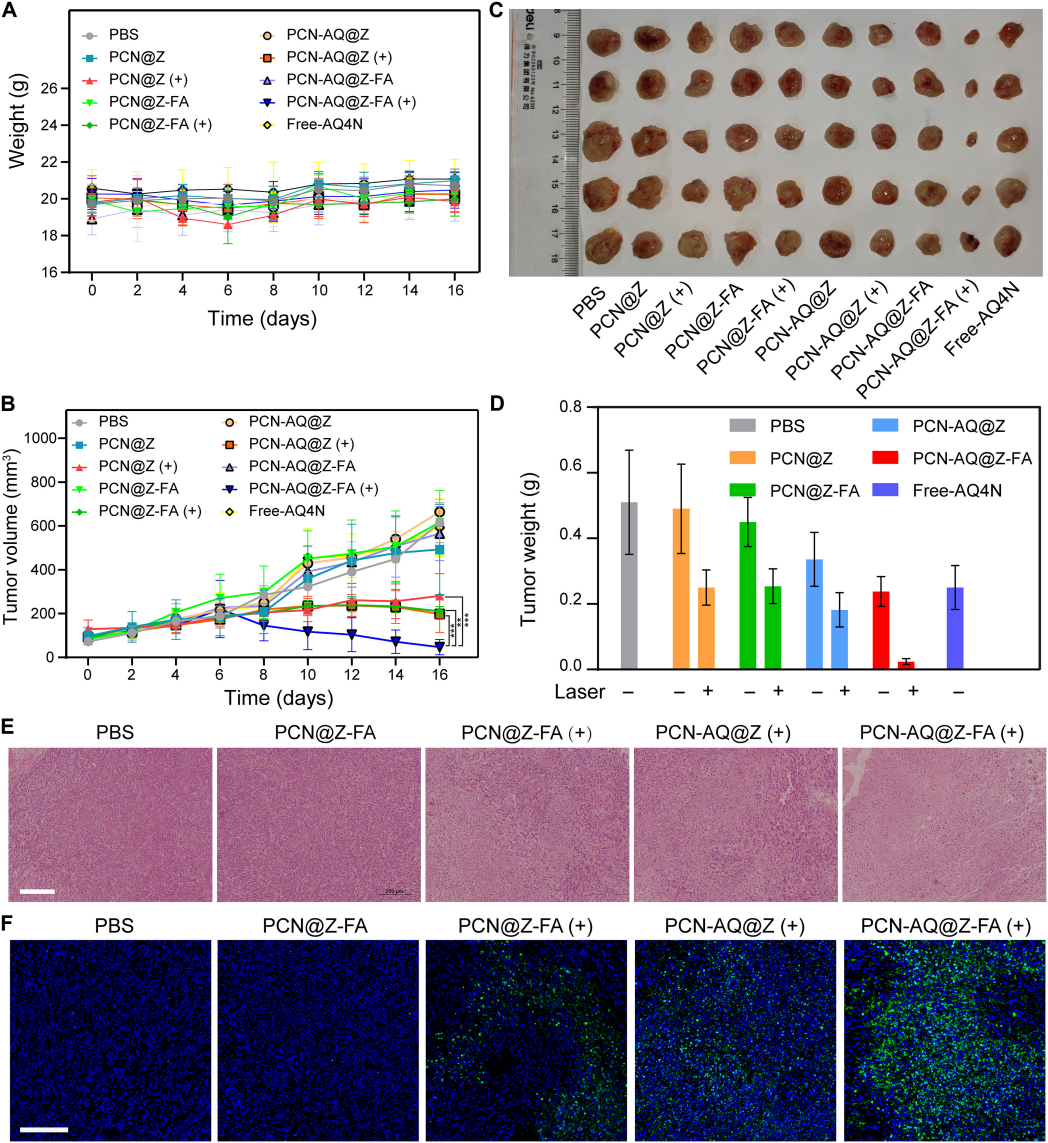
Photodynamic combined chemotherapy inhibited tumor growth. (A) Temporal changes in body weight of BALB/c mice under different treatments. The data are presented as the mean ± SD (*n* = 6). (B) Tumor growth curves after different treatments. The data are presented as the mean ± SD (*n* = 6). *P < 0.05, **P < 0.01, ***P < 0.001 (independent-samples *t* test). (C) Representative pictures of the tumors and (D) average tumor weights of mice on day 16 in the different treatment groups. The data are presented as the mean ± SD (*n* = 5). (E) Histological assessments of tumors using hematoxylin and eosin (H&E) staining in the different treatment groups. Scale bar, 200 μm. (F) TUNEL staining of tumor sections collected from mice treated as indicated. Scale bar, 100 μm.

Next, we investigated the effectiveness of NPs in treating tumors in vivo. First, 4T1 tumor-bearing mice were treated with PBS (−/+), Free-TCPP (−/+), and PCN@Z (−/+). Under the same laser irradiation conditions (100 mW cm^−2^, 10 min), tumor-bearing mice treated with Free-TCPP (+) and PCN@Z (+) exhibited a similar delay in tumor growth (Fig. S23). The results indicated the similar ROS generation capacity of Free-TCPP and PCN@Z, which is consistent with the results of DPBF and DCFH-DA assay. Next, to further explore the in vivo synergistic therapeutic efficacy of PCN-AQ@Z-FA NPs, 50 tumor-bearing mice were randomly divided into 10 groups, with 5 mice in each group. The groups were treated with PBS, PCN@Z (−/+), PCN@Z-FA (−/+), PCN-AQ@Z (−/+), PCN-AQ@Z-FA (−/+), and Free-AQ4N, respectively. The animal schedule table during the in vivo experiment is presented in Fig. S24. The results showed that the mice in all groups had slight increases in weight within 16 days, suggesting the low toxicity of the NPs (Fig. 8A). As shown in Fig. 8B, the tumor volumes in the groups of PBS and PCN@Z increased rapidly. In contrast, the tumor inhibition rates were 54.3% and 67.8% in the groups treated with PCN@Z (+) or PCN-AQ@Z (+), because of the antitumor results of PDT or PDT/chemo-combined therapy. Note that the antitumor inhibition result reached 92.4% in the group of PCN-AQ@Z-FA (+), indicating that an enhanced tumor-targeting ability during PDT/chemo-combined therapy plays an important role in cancer therapy.

After 16 days of treatment, all mice were euthanized to harvest the tumors. The tumor weights and pictures are presented in Fig. 8C and D, respectively. It was evident that the PCN-AQ@Z-FA group with 640-nm light irradiation had the smallest tumor volume and lightest tumor weight, which corresponded well with the tumor growth profiles presented in Fig. 8B. Additionally, the tumors were subjected to histological examination through hematoxylin and eosin (H&E) and TdT (terminal deoxynucleotidyl transferase)-mediated dUTP nick end labeling (TUNEL) staining assay. As shown in Fig. 8E and F, the treatment with PCN-AQ@Z-FA (+) resulted in more apoptotic or necrotic tumor cells than other treatments, thus validating the remarkable therapeutic potential of the photo-irradiated PCN-AQ@Z-FA.

It is crucial to ensure that biomedical nanomotors used for clinical cancer therapy are safe for long-term use within the body. To assess the biocompatibility of PCN-AQ@Z-FA, H&E staining images of the main organs (heart, liver, spleen, lung, and kidney) were analyzed after different treatments (Fig. S25). The complete blood counts for all groups were normal (Fig. S26), and there were no conspicuous changes in the levels of serum biochemical parameters (Fig. S27). These results indicate that PCN-AQ@Z-FA is safe for in vivo use and can be considered for future biomedical applications.

## Conclusion

In summary, a ZIF-8-gated PCN nanosystem has been successfully constructed for highly effective PDT and the pH-triggered release of AQ4N. Moreover, FA-PEG was conjugated on the nanostructure for specific biorecognition capability and prolonged blood circulation time. The as-obtained nanostructures, labeled as PCN-AQ@Z-FA, encompass the benefits of (a) a porous nanostructure for effective AQ4N loading, (b) photo-induced ROS generation behavior characterized by the PCN core, (c) efficient bioreduction of AQ4N to AQ4 under a hypoxia environment for chemotherapy, and (d) the “gatekeeper” characteristic of ZIF-8 shell for the pH-triggered AQ4N release. Both in vitro and in vivo investigations have shown that the synthesized PCN-AQ@Z-FA is highly effective in treating hypoxic tumors with minimal side effects. This study highlights the potential of porphyrinic MOFs with hypoxia-activated prodrugs for precision PDT.

## Data Availability

Data will be made available on request.
