# Supplementary material for: Core–Shell Structured Metal–Organic Frameworks for pH-Triggered Combination Photodynamic/Chemotherapy-Based Cancer Treatment
Source: Biomater Res. 2025 Jan 22;29:0138. doi: 10.34133/bmr.0138 (PMC11751201; doi:10.34133/bmr.0138)
Supplement: Supplementary 1 — Figs. S1 to S27 [file bmr.0138.f1.docx]

**Supplementary Materials**

**Additional file: Fig. S1.** SEM images of PCN-222. **Fig. S2.** (A) TEM image of PCN-AQ. (B) Size distributions of PCN. (C) Size distributions of PCN-AQ. **Fig. S3.** SEM images of PCN-AQ@Z. **Fig. S4.** Dynamic light scattering (DLS) patterns of PCN, PCN@Z and PCN@Z-FA NPs. **Fig. S5.** UV-vis-NIR absorption of PCN-AQ@Z-FA. **Fig. S6**. N_2_ sorption isotherms and pore size distributions of PCN, PCN-AQ and PCN-AQ@Z NPs. **Fig. S7**. AQ4N release profiles of PCN-AQ at different pH conditions. **Fig. S8.** Standard curve of different concentrations of TCPP and its absorbance at 652 nm. **Fig. S9.** The absorption spetra of different groups: (A) Free-TCPP under laser irradiation. (B) The mixed solution of DPBF and Free-TCPP without laser irradiation. (C) The mixed solution of DPBF and Free-TCPP under laser irradiation. (D) PCN@Z-FA under laser irradiation. (E) The mixed solution of DPBF and PCN@Z-FA without laser irradiation. (F) The mixed solution of DPBF and PCN@Z-FA under laser irradiation. **Fig. S10.** Flow cytometry analysis of MCF-7 cells treated with PBS, Free-AQ4N, PCN-AQ@Z and PCN-AQ@Z-FA. **Fig. S11.** CLSM images of 4T1 cells treated with PBS, Free-AQ4N, PCN-AQ@Z and PCN-AQ@Z-FA at 37°C for 4 h. 4T1 were stained with Hoechst (blue). Scale bar 20 µm. **Fig. S12.** CLSM images of A549 cells treated with PCN-AQ@Z and PCN-AQ@Z-FA (Blue represents Hoechst, red represents AQ4N). Scale bar: 10 μm. **Fig. S13.** CLSM images of MCF-7 cells after incubation with PCN-AQ@Z-FA for 0.5 h, 1 h and 2 h. The endo/lysosomes were stained by Lyso-Tracker Green. Scale bar, 20 μm. **Fig. S14.** CLSM images of MCF-7 cells after incubation with PCN-AQ@Z-FA (+) for 0.5 h and 4 h. PCN-AQ@Z-FA successfully escaped from endo/lysosomes proved by the separation of green and red fluorescence. The endo/lysosomes were stained by Lyso-Tracker Green. Scale bar, 50 μm. **Fig. S15.** CLSM analysis of ROS generation in 4T1 cells treated with PBS, Free-TCPP, PCN@Z and PCN@Z-FA. Scare bars: 50 µm. **Fig. S16.** The intracellular behaviors of PCN-AQ@Z-FA and Free AQ4N under normoxia or hypoxia conditions. Scale bar, 10 μm. **Fig. S17**. Viabilities of 4T1 cells treated with different concentrations of PCN-AQ@Z-FA with or without light irradiation. **Fig. S18.** Viability of MCF-7 cells treated with PCN-AQ@Z-FA following different irradiation times. **Fig. S19.** CCK-8 assay of MCF-7 cells after incubating with PCN-AQ@Z-FA (without laser irradiation) at normoxia/hypoxia environment. **Fig. S20**. CCK-8 assay was performed to evaluate 4T1 cell viability upon different treatments. **Fig. S21.** CLSM images of MCF-7 cells treated with PCN@Z-FA and Free-AQ4N (Green represents live cells, red represents dead cells). Scale bar: 50 μm. **Fig. S22.** Flow cytometry analysis of MCF-7 cells treated with PCN@Z, PCN@Z-FA and Free AQ4N. **Fig. S23**. (A)Temporal changes in body weight of BALB/c mice under different treatments. The data are presented as the mean ± s.e.m. (n = 6). (B) Tumor growth curves after different treatments. The data are presented as the mean ± s.e.m. (n = 6). **P* < 0.05, ***P* < 0.01, ****P* < 0.001 (independent sample t-test). (C) Representative pictures of the tumors and (D) average tumor weights of mice at day 15 in the different treatment groups. The data are presented as the mean ± s.e.m. (n = 5). **Fig. S24**. The animal schedule table during the in vivo experiment. **Fig. S25.** Histological assessments of different organs using H&E staining in the different treatment groups. Scale bar: 200 μm. **Fig. S26**. Whole blood analyses from tumor-bearing mice after different treatments. The whole blood indicators include red blood cells (RBC), hemoglobin (HGB), red blood distribution width (RDW), hematocrit (HCT), mean corpuscular volume (MCV), mean corpuscular hemoglobin (MCH), white blood cells (WBC), platelets (PLT), platelet distribution width (PDW), neutrophilic (NEUT), lymphocyte (LYMPH) and monocyte (MONO). **Fig. S27**. Serum biochemistry analysis of tumor-bearing mice after various treatments to investigate the potential systemic toxicity. The serum biochemical indexes include alanine aminotransferase (ALT), aspartate aminotransferase (AST), albumin (ALB), urea nitrogen (BUN), creatinine (CREA), Total Protein (TP).


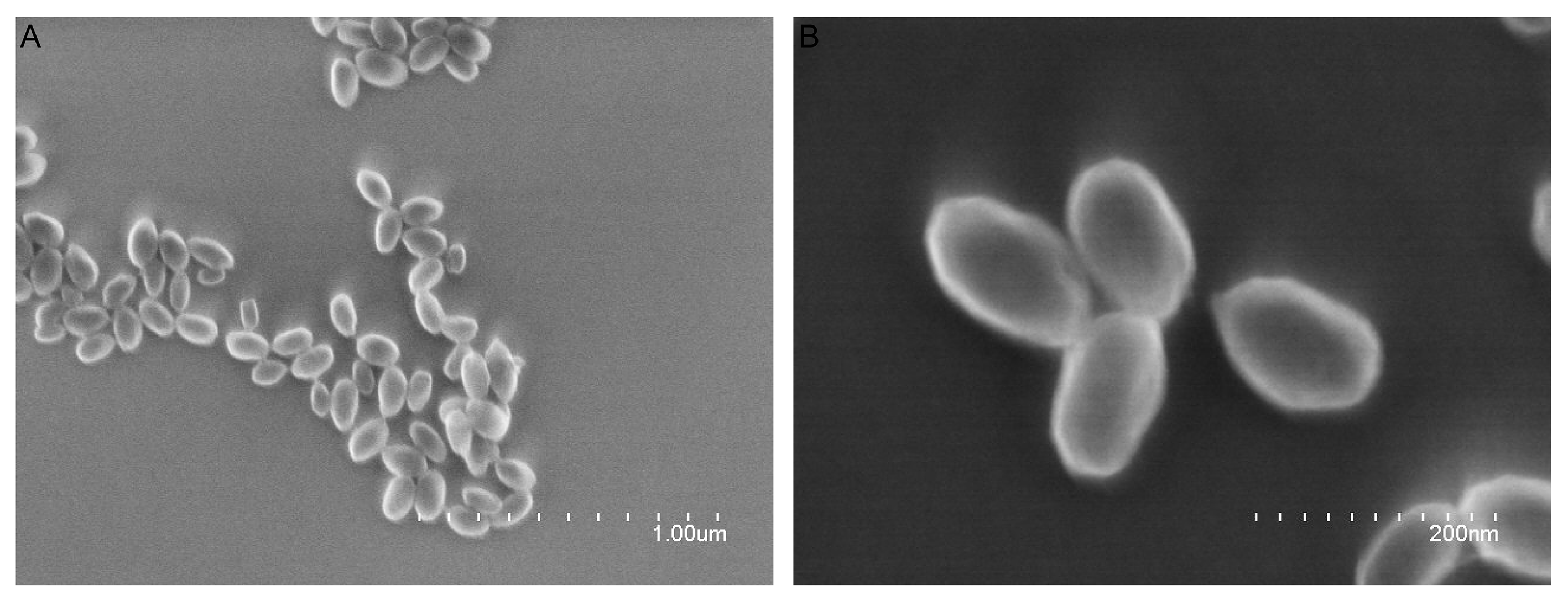


**Fig. S1.** SEM images of PCN.


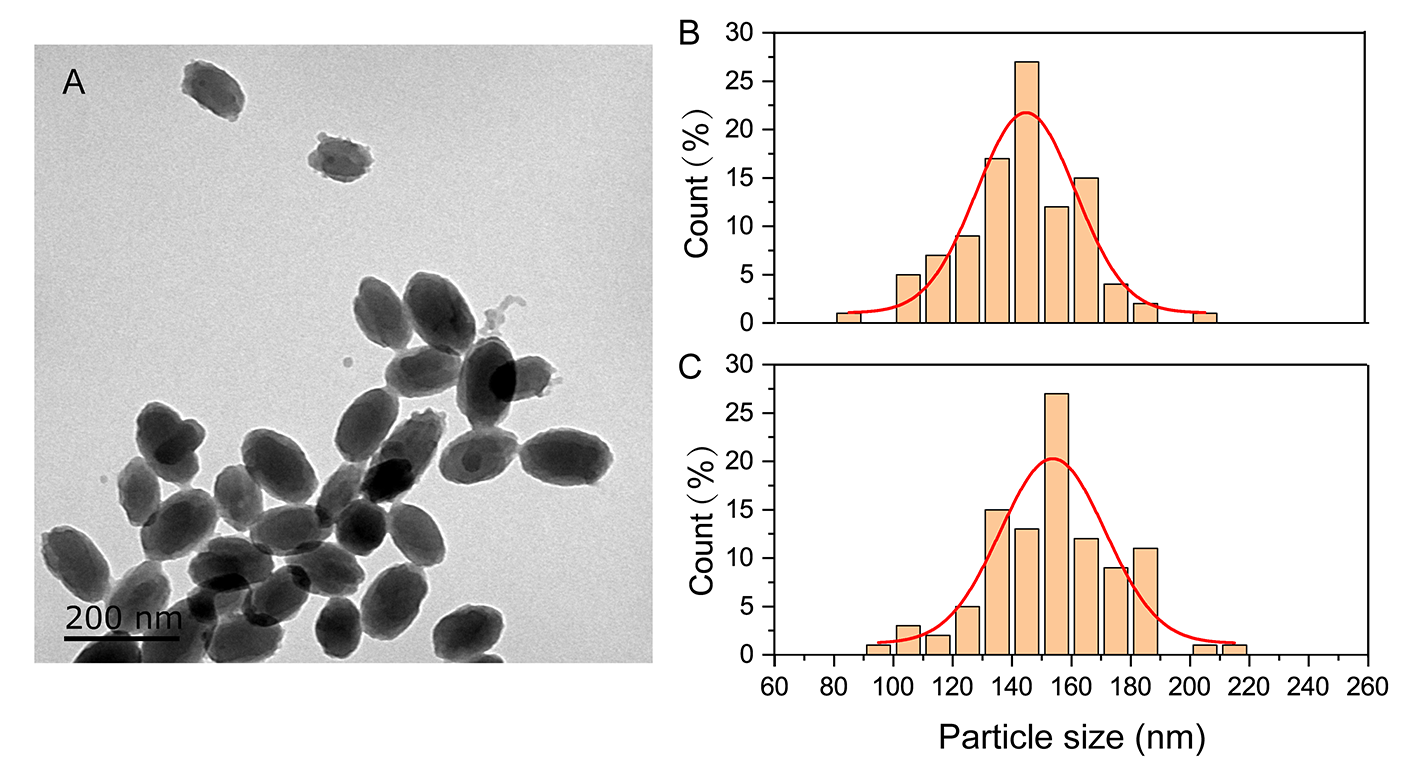


**Fig. S2.** (A) TEM image of PCN-AQ. (B) Size distributions of PCN. (C) Size distributions of PCN-AQ.


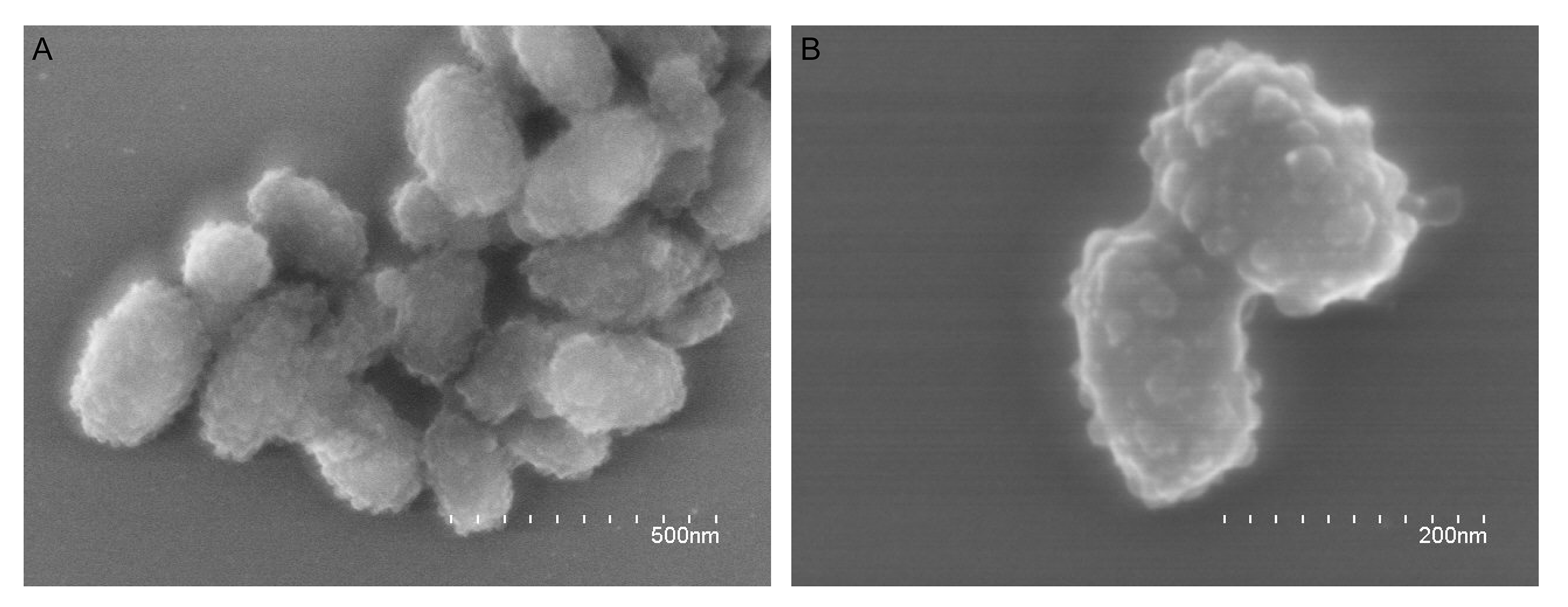


**Fig. S3.** SEM images of PCN-AQ@Z.


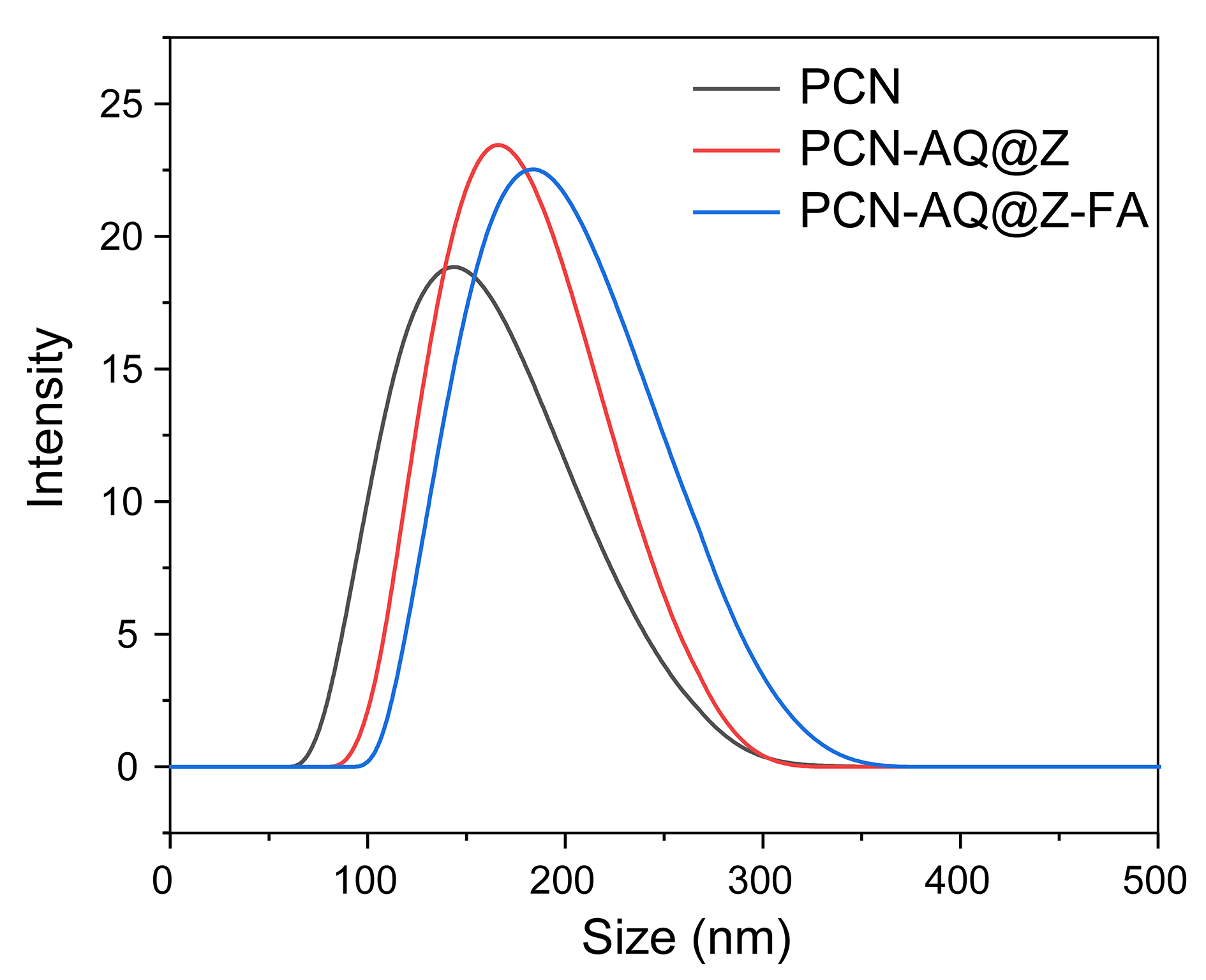


**Fig. S4.** Dynamic light scattering (DLS) patterns of PCN, PCN-AQ@Z and PCN-AQ@Z-FA NPs.


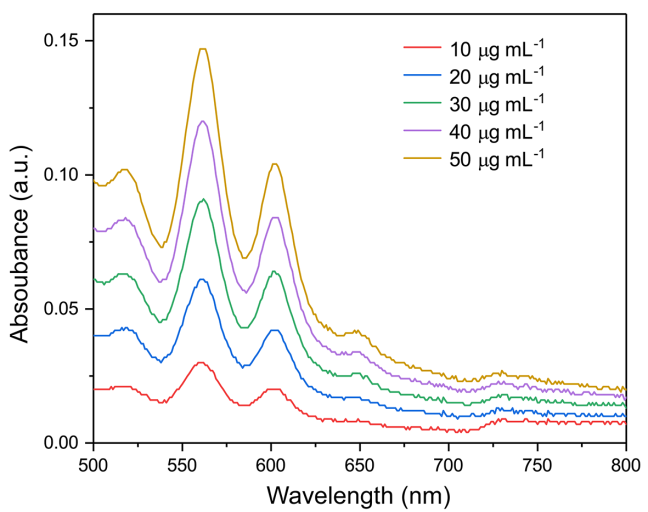


**Fig. S5.** UV-vis-NIR absorption of PCN-AQ@Z-FA.


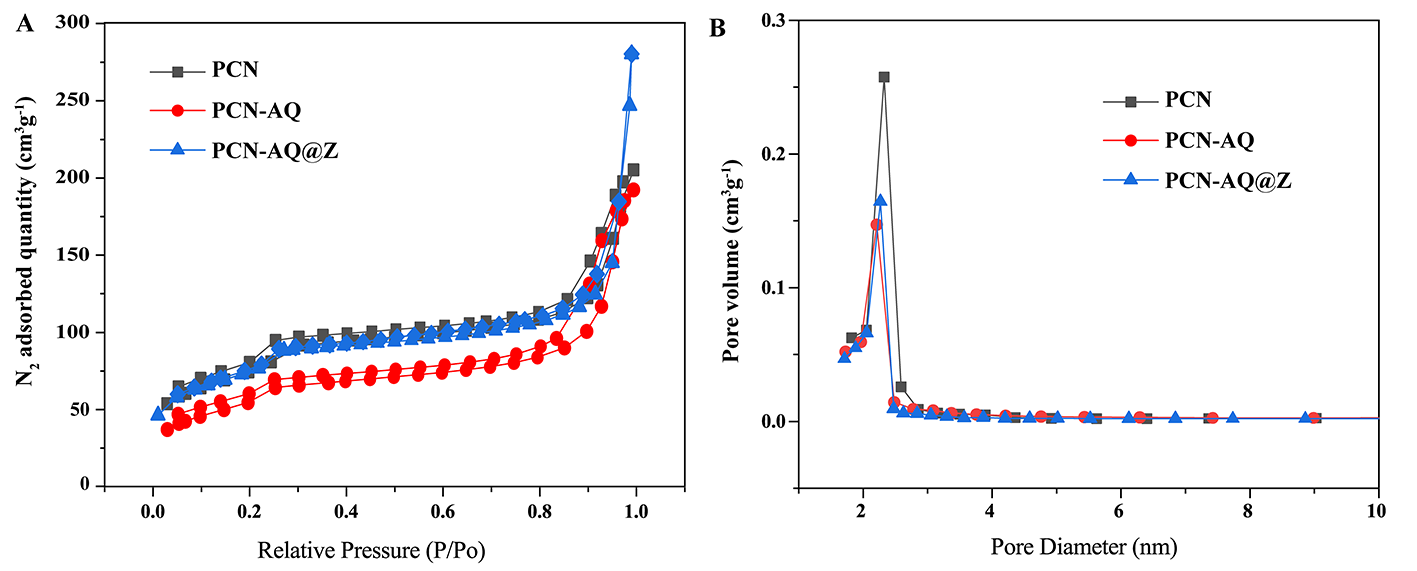


**Fig. S6.** N_2_ sorption isotherms and pore size distributions of PCN, PCN-AQ and PCN-AQ@Z NPs.


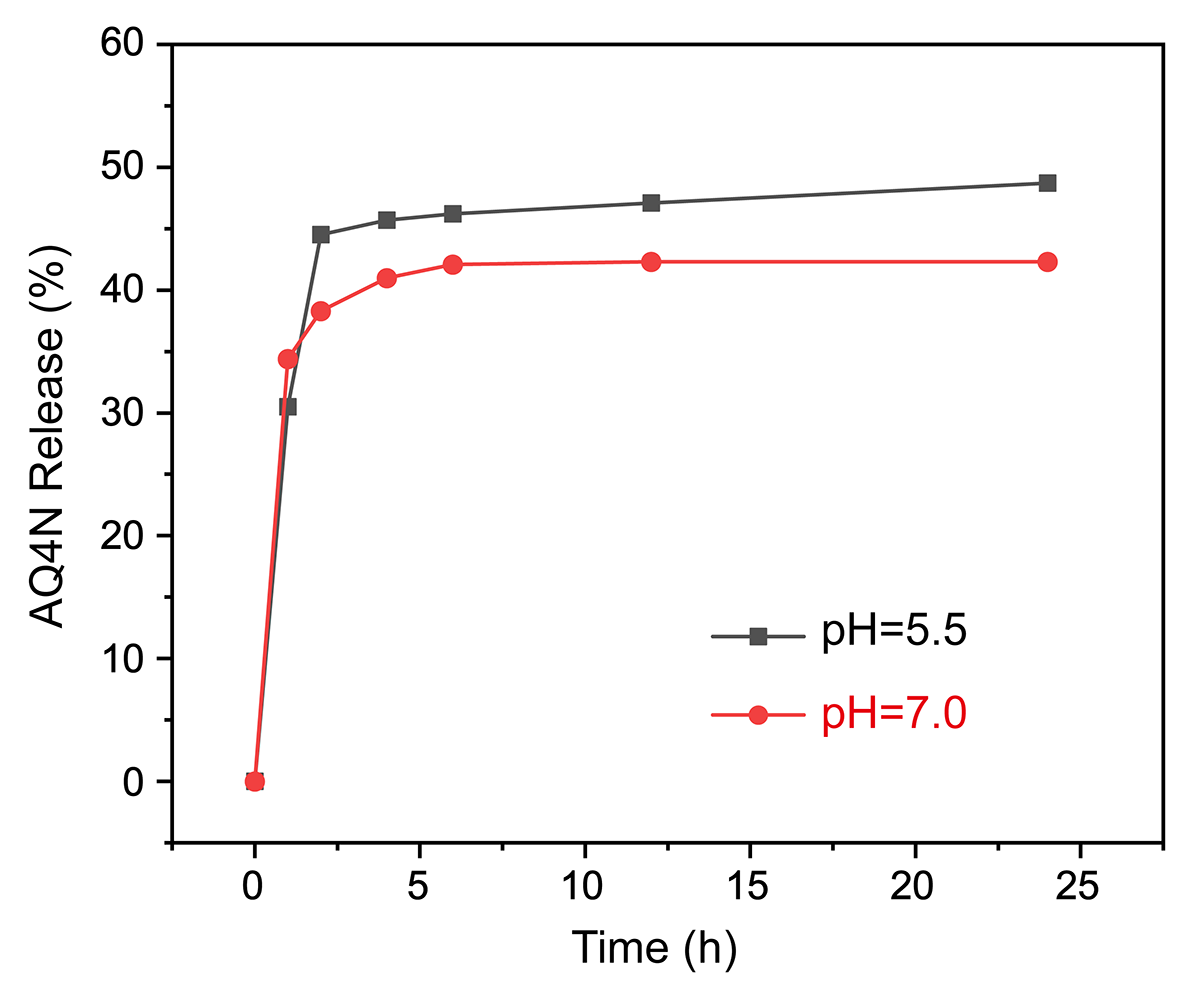


**Fig. S7.** AQ4N release profiles of PCN-AQ at different pH conditions.

**
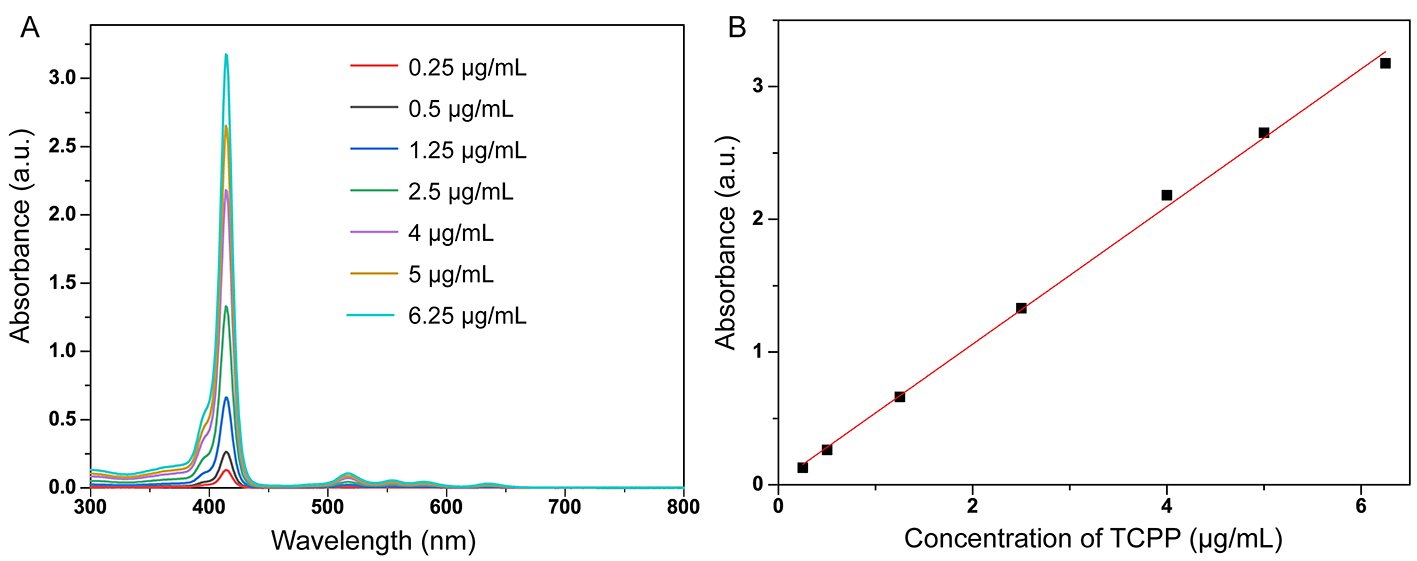
**

**Fig. S8.** Standard curve of different concentrations of TCPP and its absorbance at 414 nm.

**
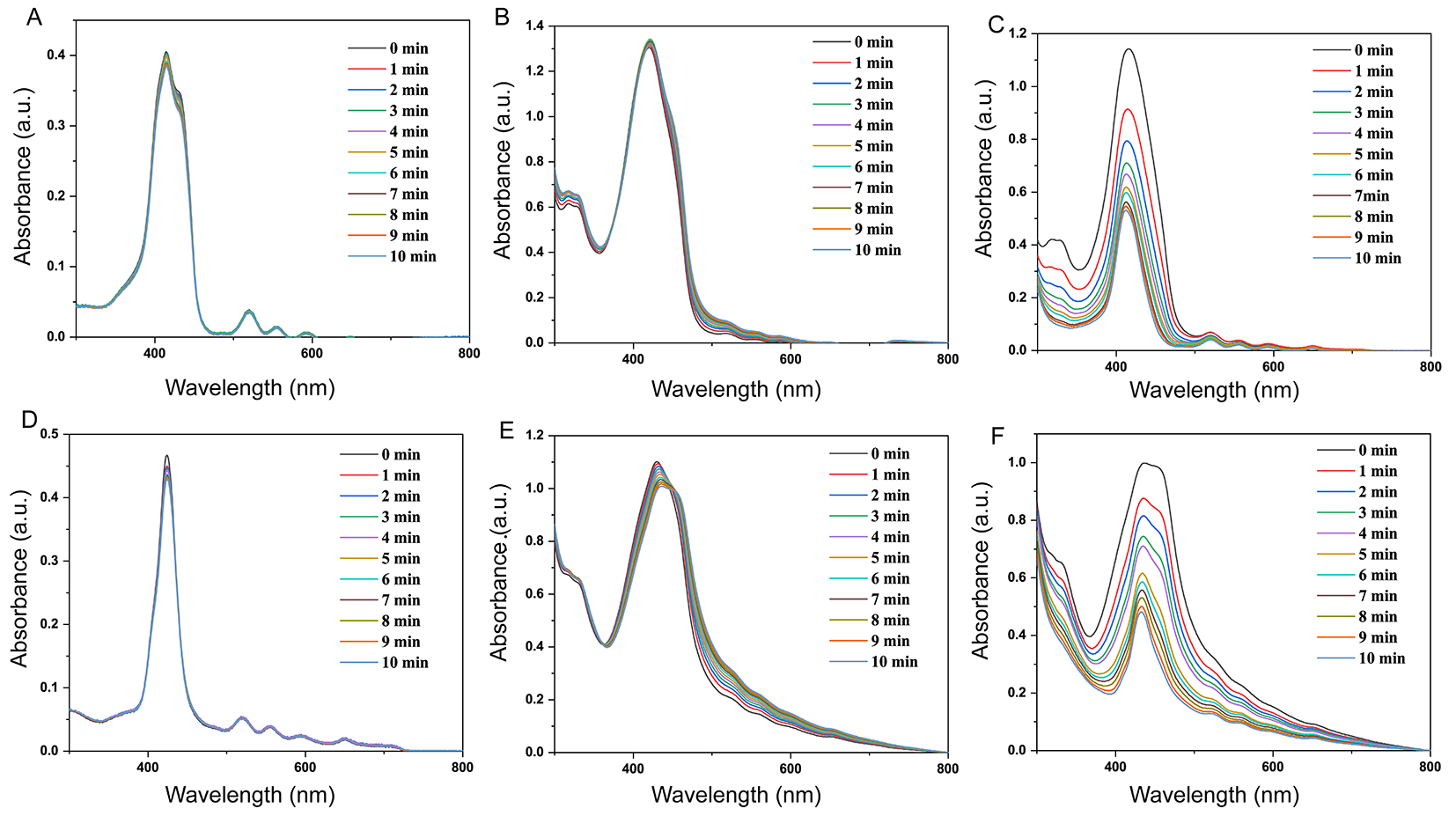
**

**Fig. S9.** The absorption spectra of different groups: (A) Free-TCPP under laser irradiation. (B) The mixed solution of DPBF and Free-TCPP without laser irradiation. (C) The mixed solution of DPBF and Free-TCPP under laser irradiation. (D) PCN@Z-FA under laser irradiation. (E) The mixed solution of DPBF and PCN@Z-FA without laser irradiation. (F) The mixed solution of DPBF and PCN@Z-FA under laser irradiation.


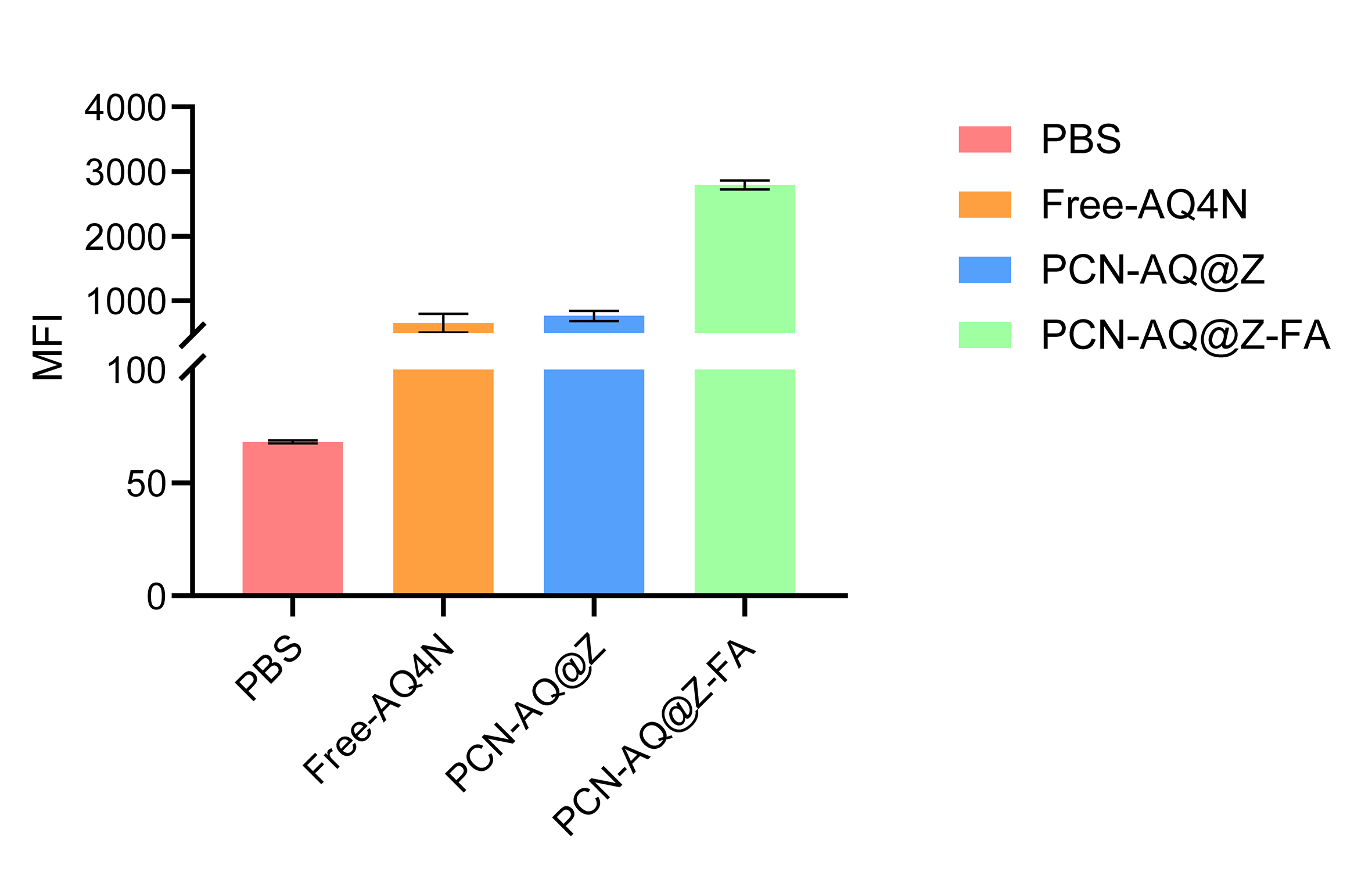


**Fig. S10.** Flow cytometry analysis of MCF-7 cells treated with PBS, Free-AQ4N, PCN-AQ@Z and PCN-AQ@Z-FA.


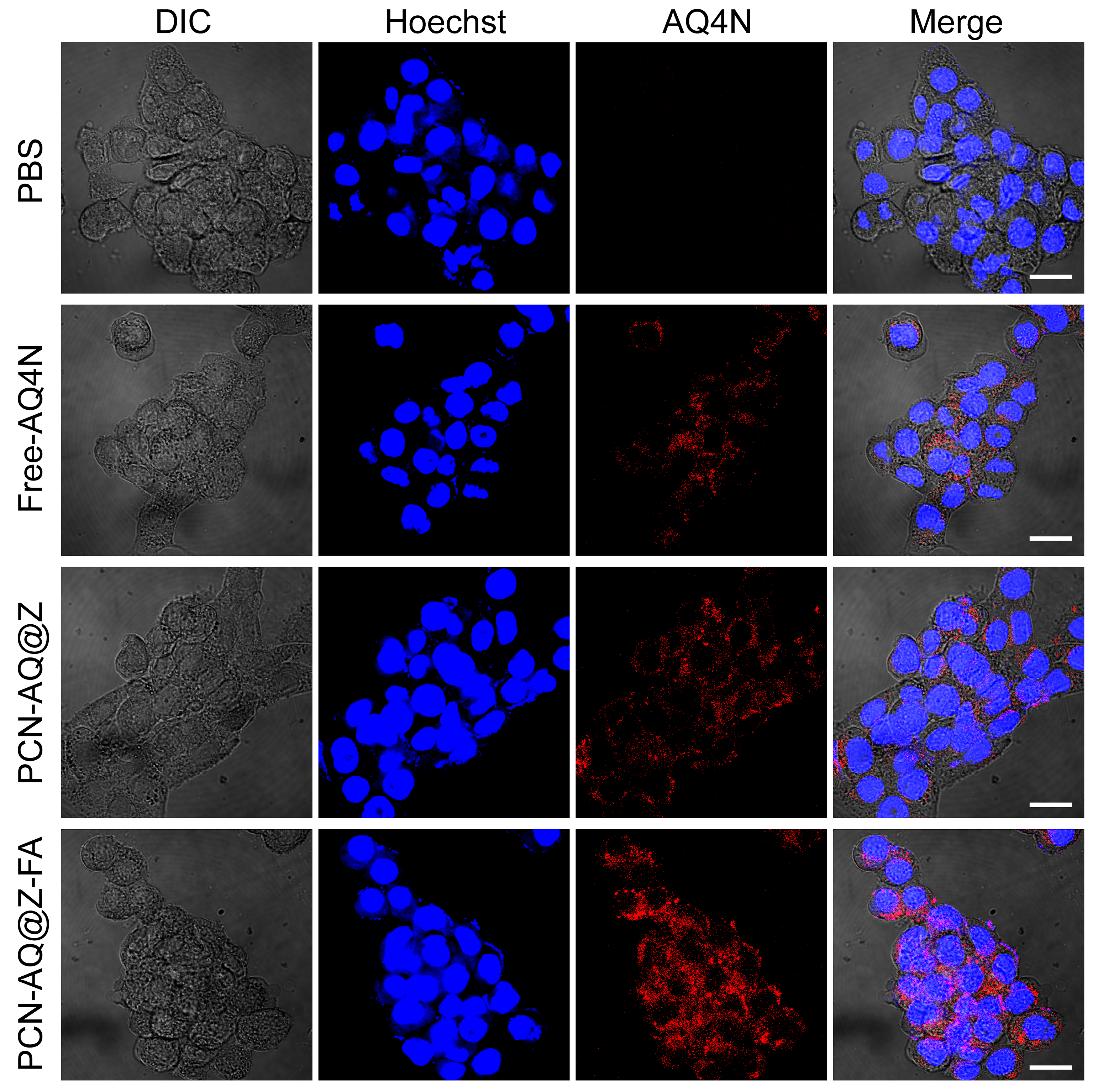


**Fig. S11.** CLSM images of 4T1 cells treated with PBS, Free-AQ4N, PCN-AQ@Z and PCN-AQ@Z-FA at 37°C for 4 h. 4T1 were stained with Hoechst (blue). Scale bar 20 µm.


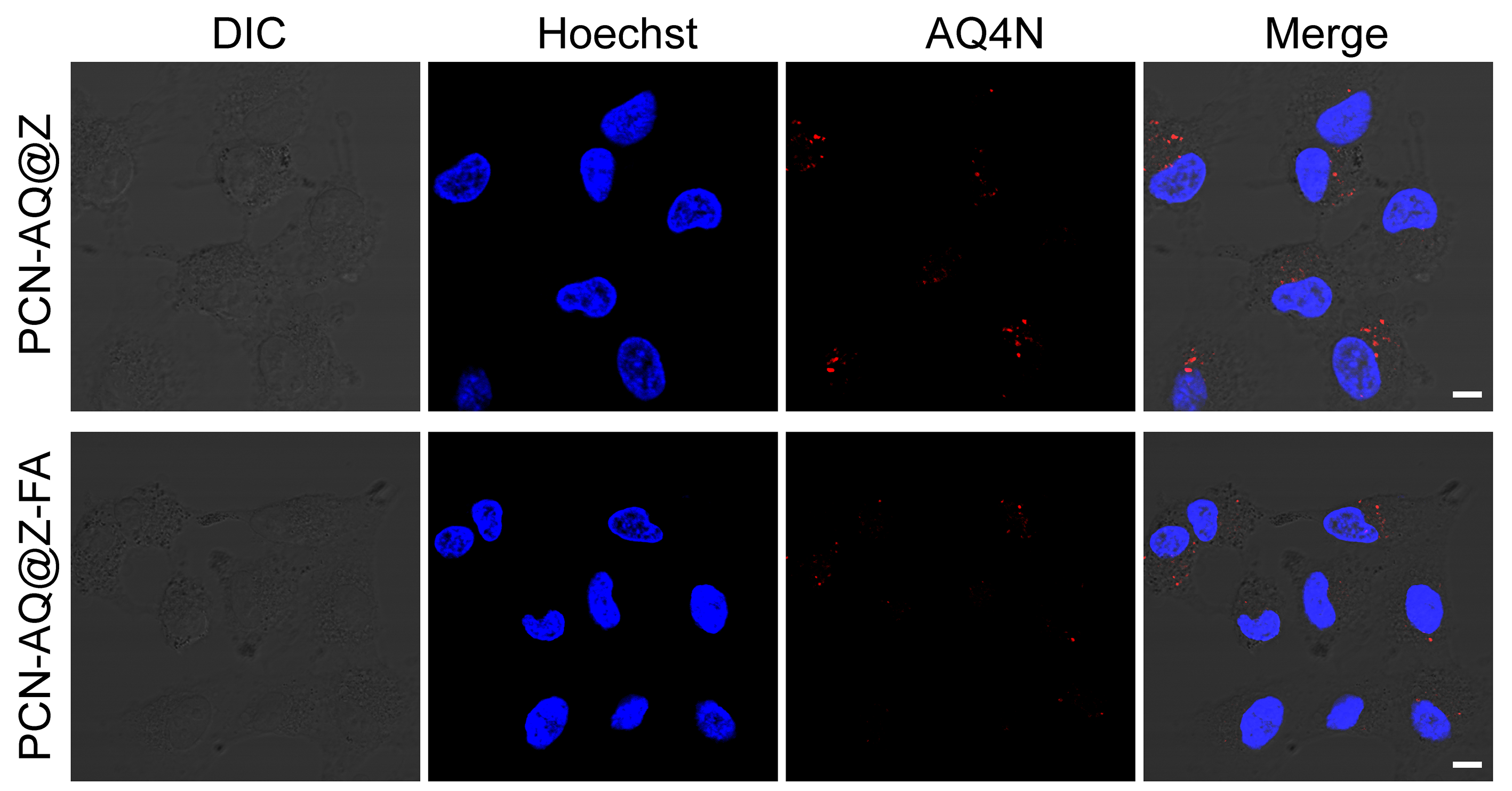


**Fig. S12.** CLSM images of A549 cells treated with PCN-AQ@Z and PCN-AQ@Z-FA (Blue represents Hoechst, red represents AQ4N). Scale bar: 10 μm.


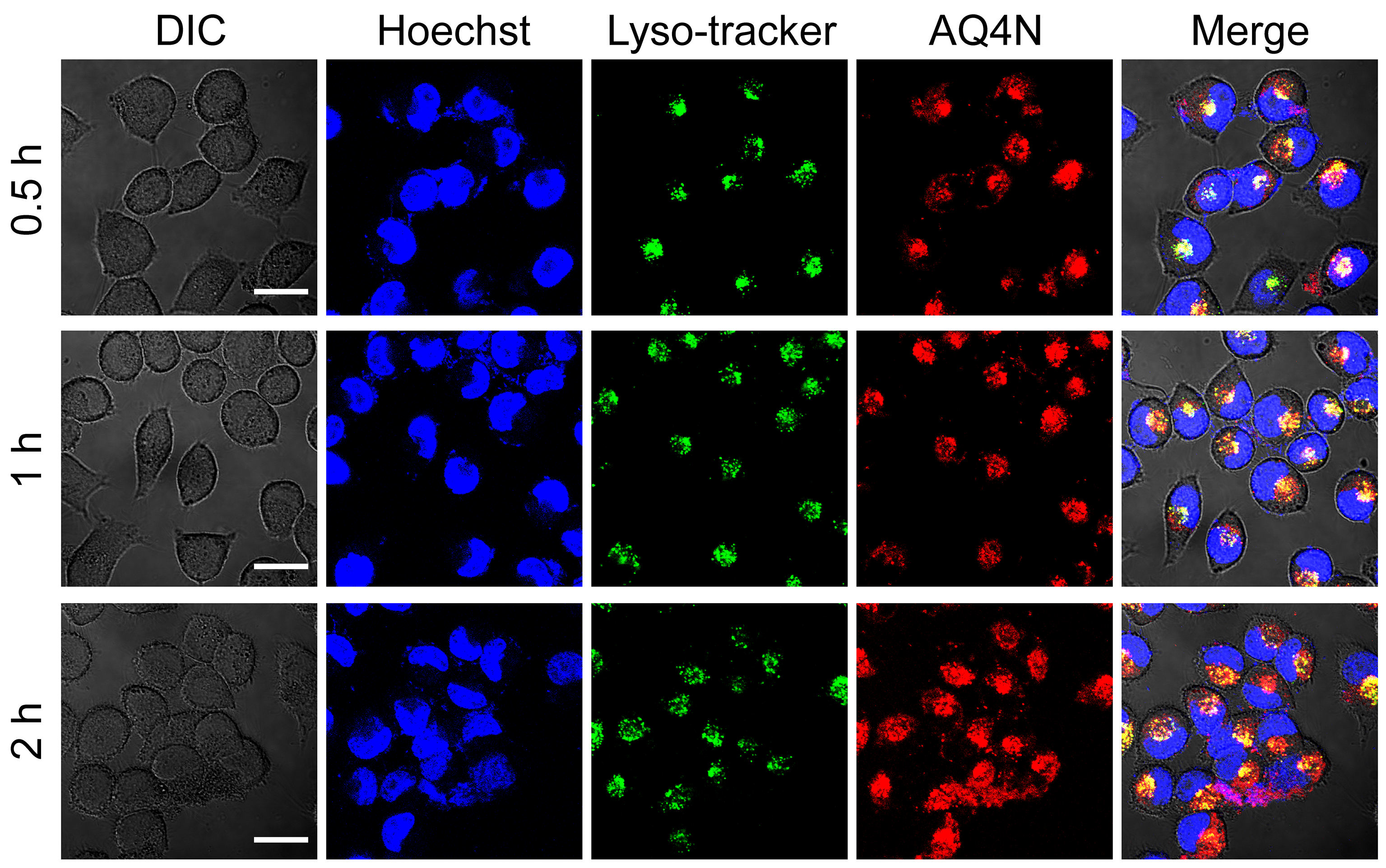


**Fig. S13.** CLSM images of MCF-7 cells after incubation with PCN-AQ@Z-FA for 0.5 h, 1 h and 2 h. The endo/lysosomes were stained by Lyso-Tracker Green. Scale bar, 20 μm.


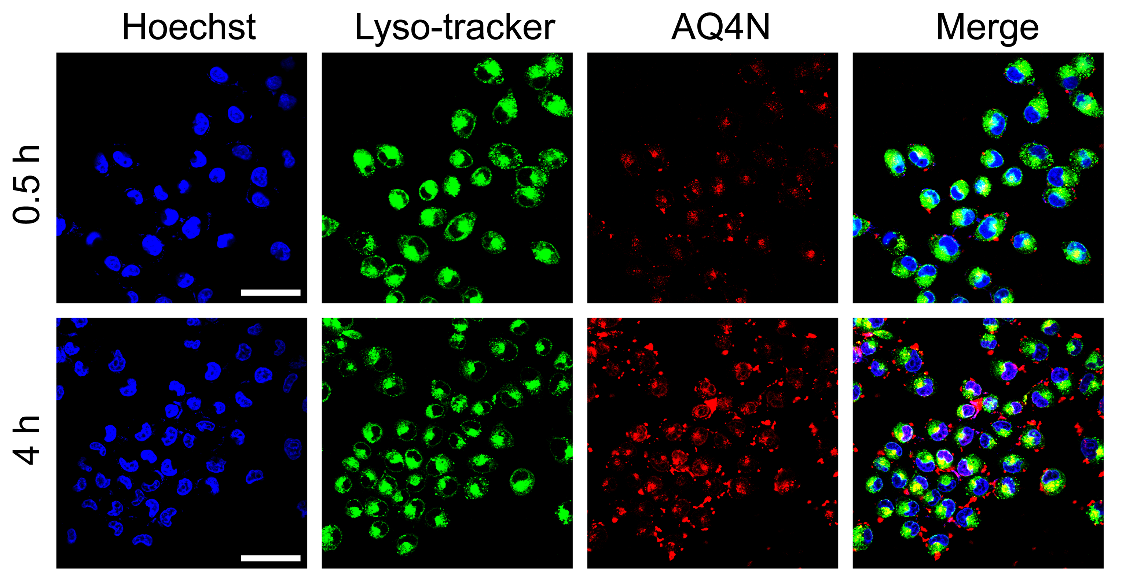


**Fig. S14**. CLSM images of MCF-7 cells after incubation with PCN-AQ@Z-FA (+) for 0.5 h and 4 h. PCN-AQ@Z-FA successfully escaped from endo/lysosomes proved by the separation of green and red fluorescence. The endo/lysosomes were stained by Lyso-Tracker Green. Scale bar, 50 μm.


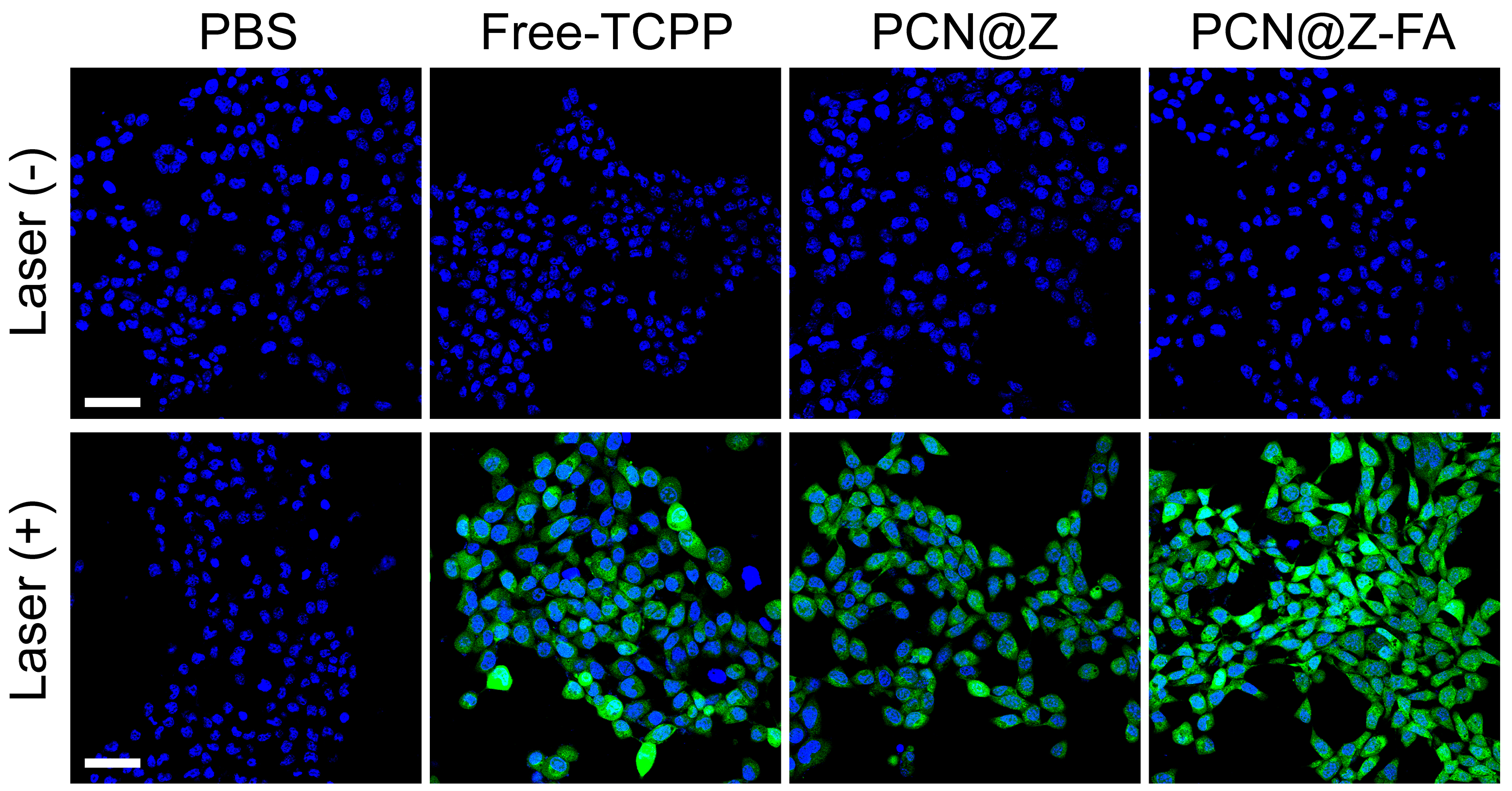


**Fig. S15.** CLSM analysis of ROS generation in 4T1 cells treated with PBS, Free-TCPP, PCN@Z and PCN@Z-FA. Scare bars: 50 µm.


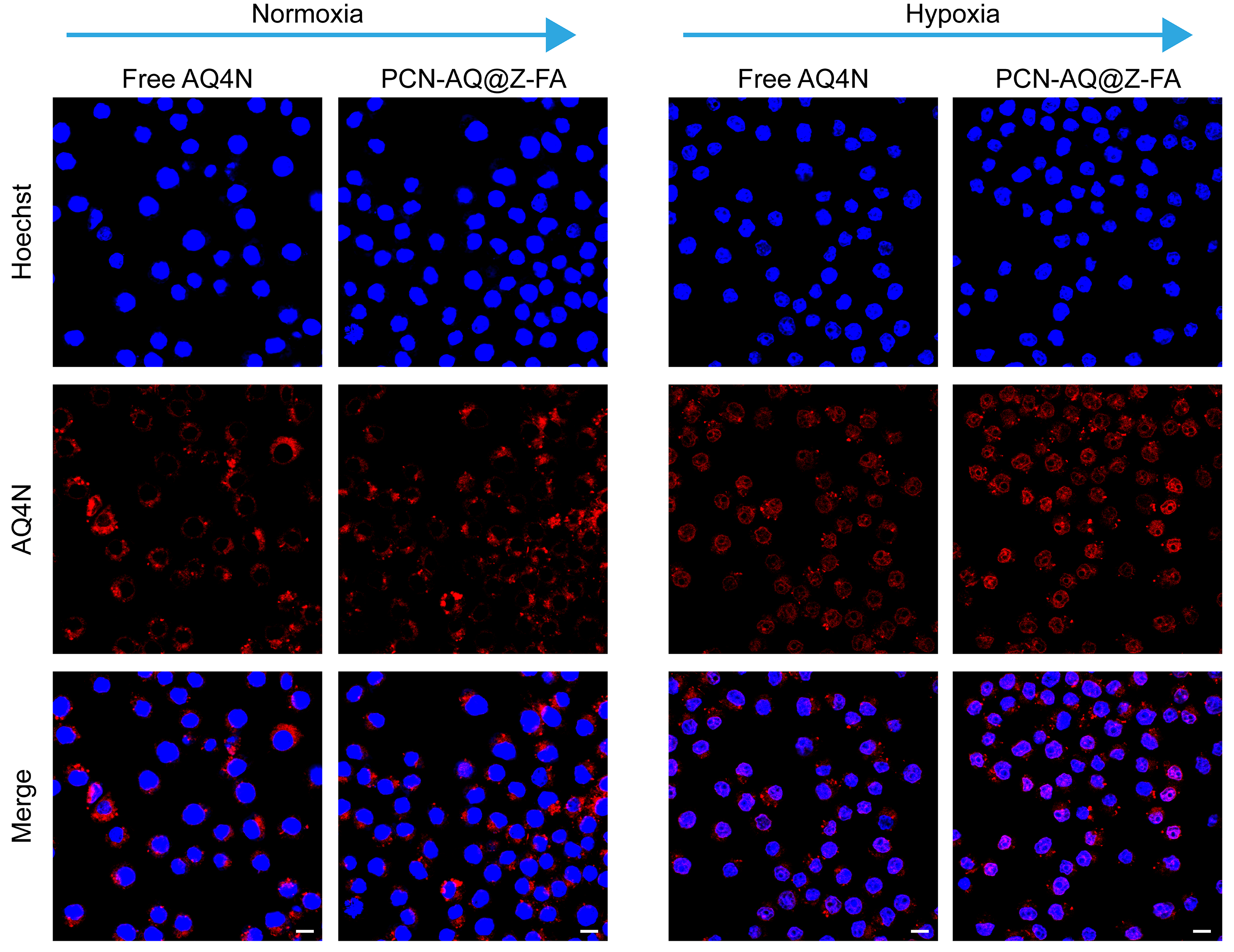


**Fig. S16.** The intracellular behaviors of PCN-AQ@Z-FA and Free AQ4N under normoxia or hypoxia conditions. Scale bar, 10 μm.

**
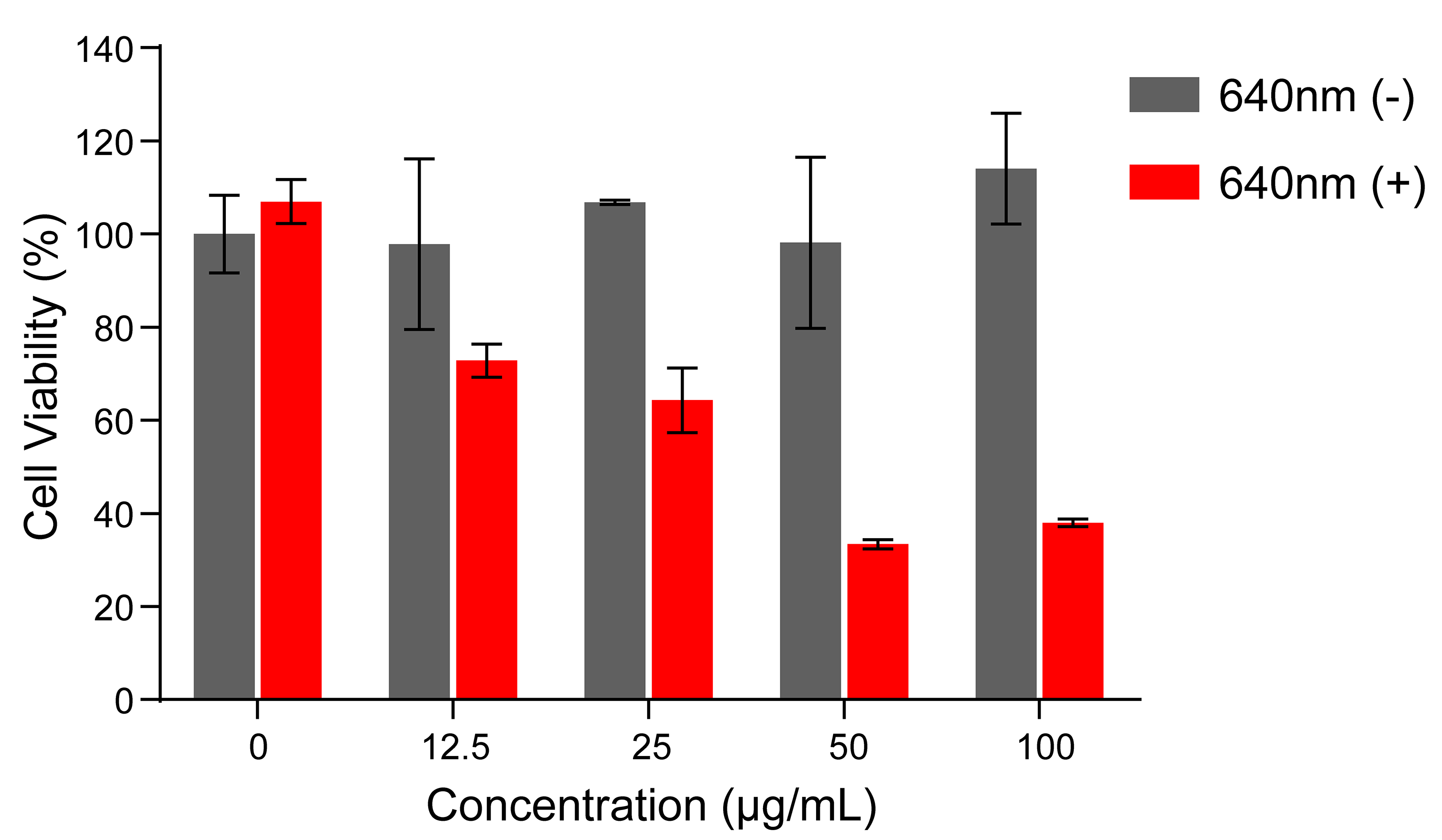
**

**Fig. S17**. Viabilities of 4T1 cells treated with different concentrations of PCN-AQ@Z-FA with or without laser irradiation.


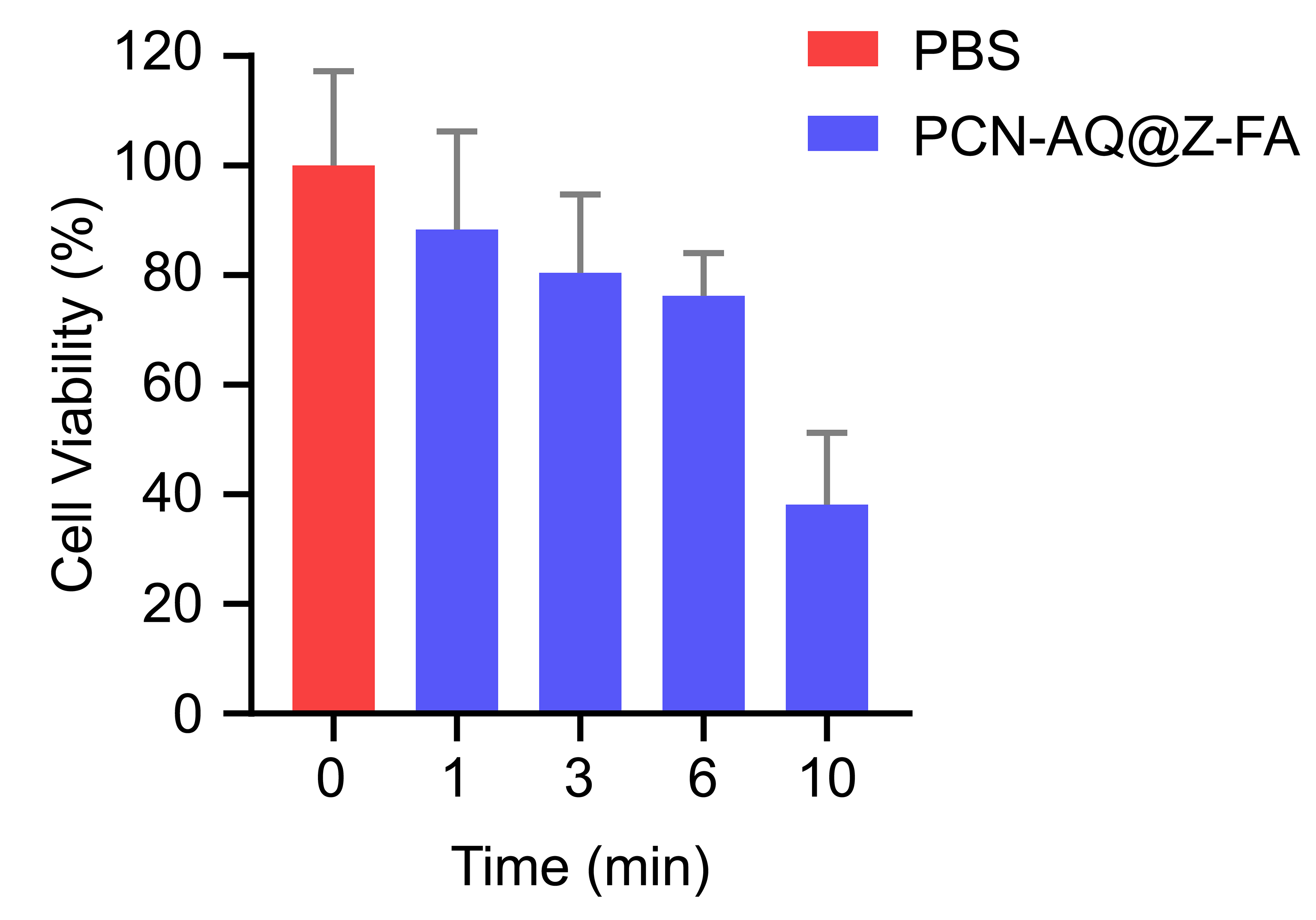


**Fig. S18**. Viability of MCF-7 cells treated with PCN-AQ@Z-FA following different irradiation times.


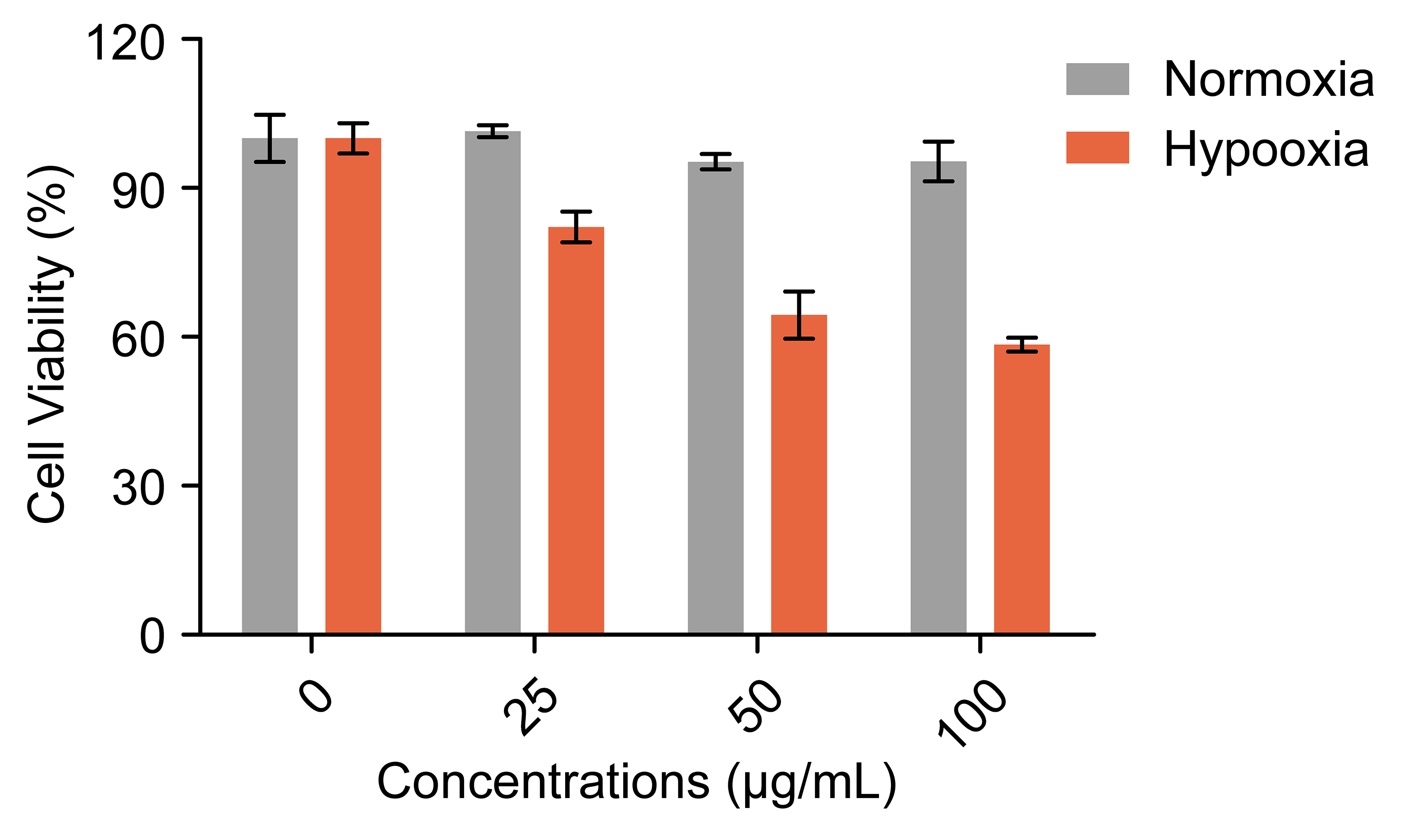


**Fig. S19**. CCK-8 assay of MCF-7 cells after incubating with PCN-AQ@Z-FA (without laser irradiation) at normoxia/hypoxia environment.

**
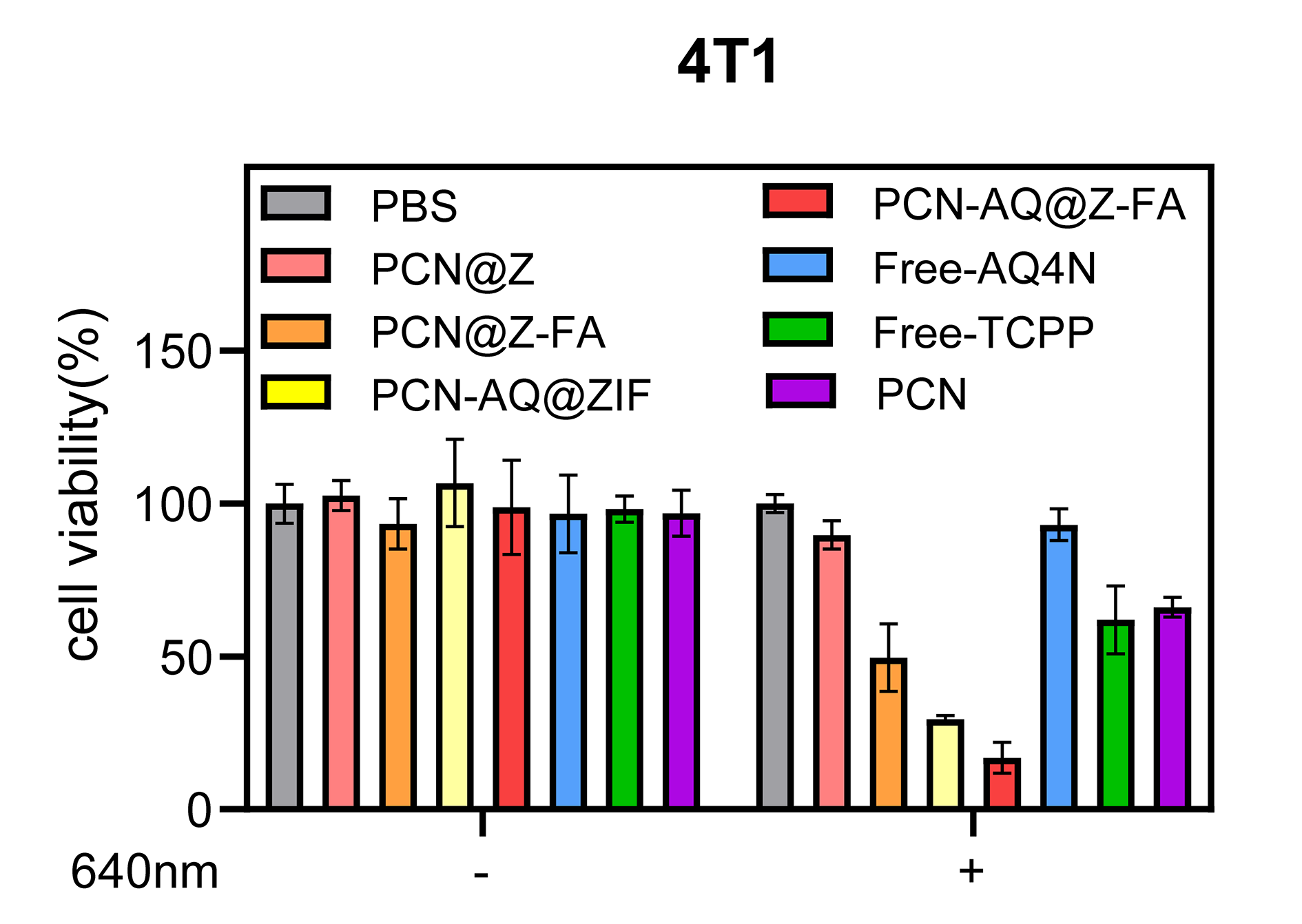
**

**Fig. S20**. CCK-8 assay was performed to evaluate 4T1 cell viability upon different treatments.


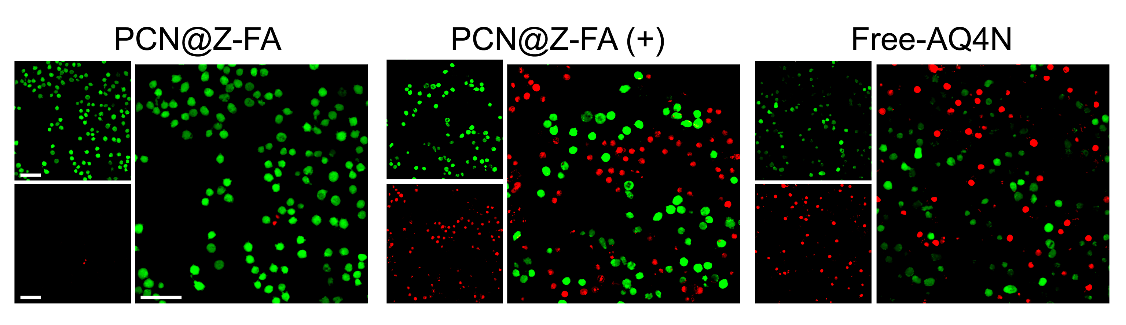


**Fig. S21.** CLSM images of MCF-7 cells treated with PCN@Z-FA and Free-AQ4N (Green represents live cells, red represents dead cells). Scale bar: 50 μm.


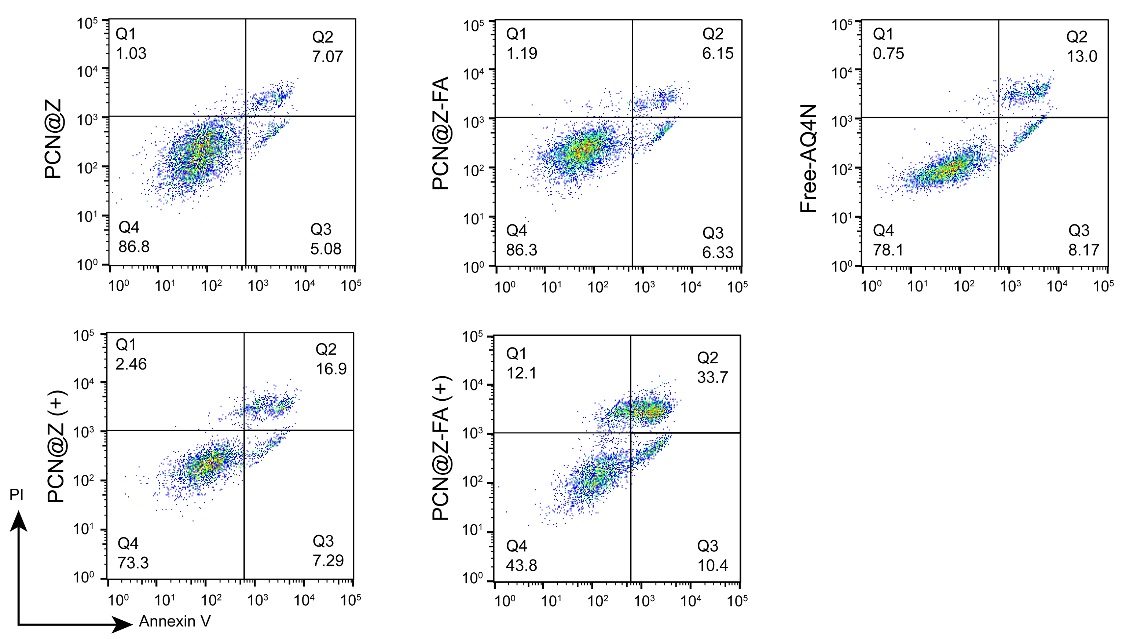


**Fig. S22.** Flow cytometry analysis of MCF-7 cells treated with PCN@Z, PCN@Z-FA and Free AQ4N.


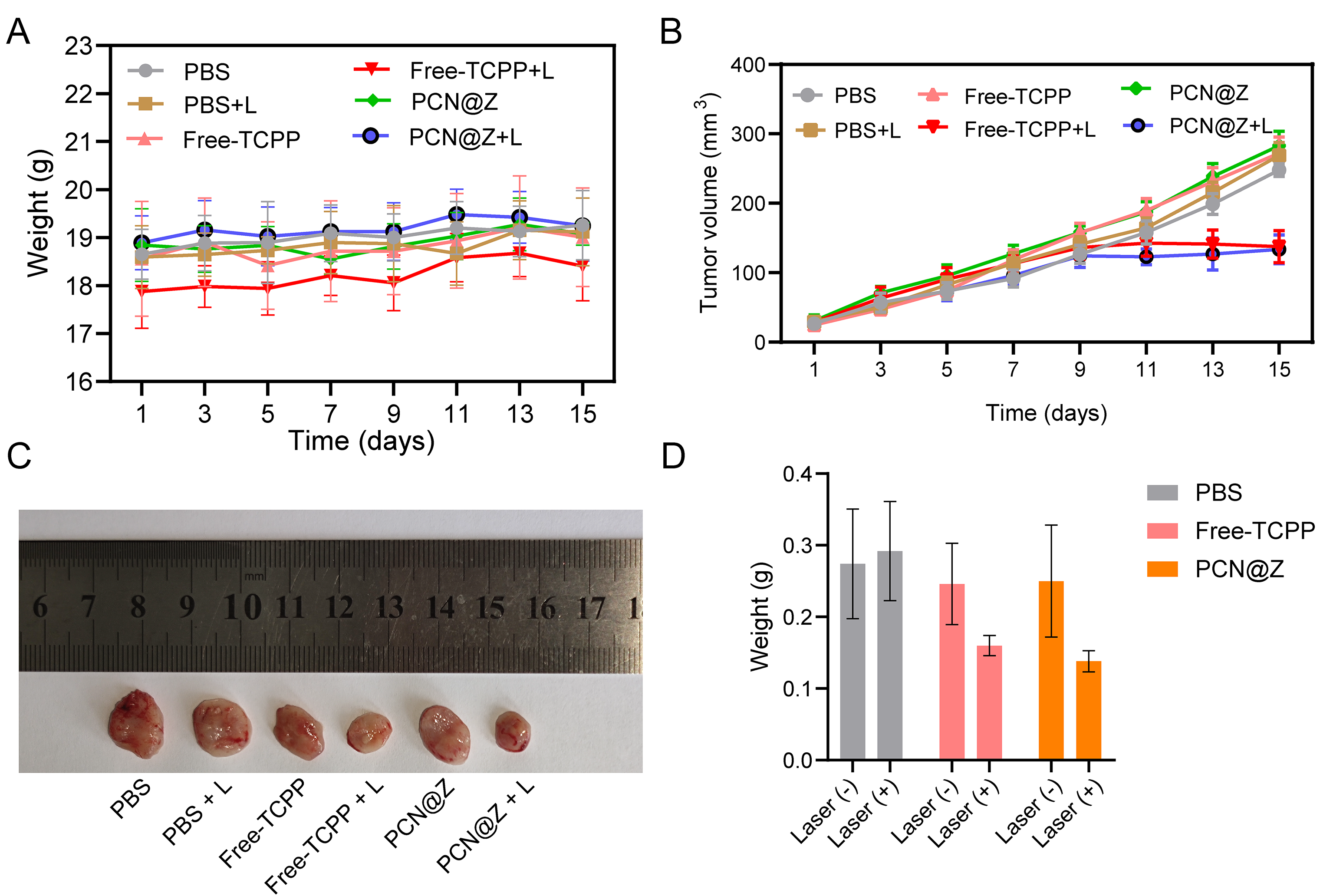


**Fig. S23.** (A)Temporal changes in body weight of BALB/c mice under different treatments. The data are presented as the mean ± s.e.m. (n = 6). (B) Tumor growth curves after different treatments. The data are presented as the mean ± s.e.m. (n = 6). **P* < 0.05, ***P* < 0.01, ****P* < 0.001 (independent sample t-test). (C) Representative pictures of the tumors and (D) average tumor weights of mice on day 15 in the different treatment groups. The data are presented as the mean ± s.e.m. (n = 5).


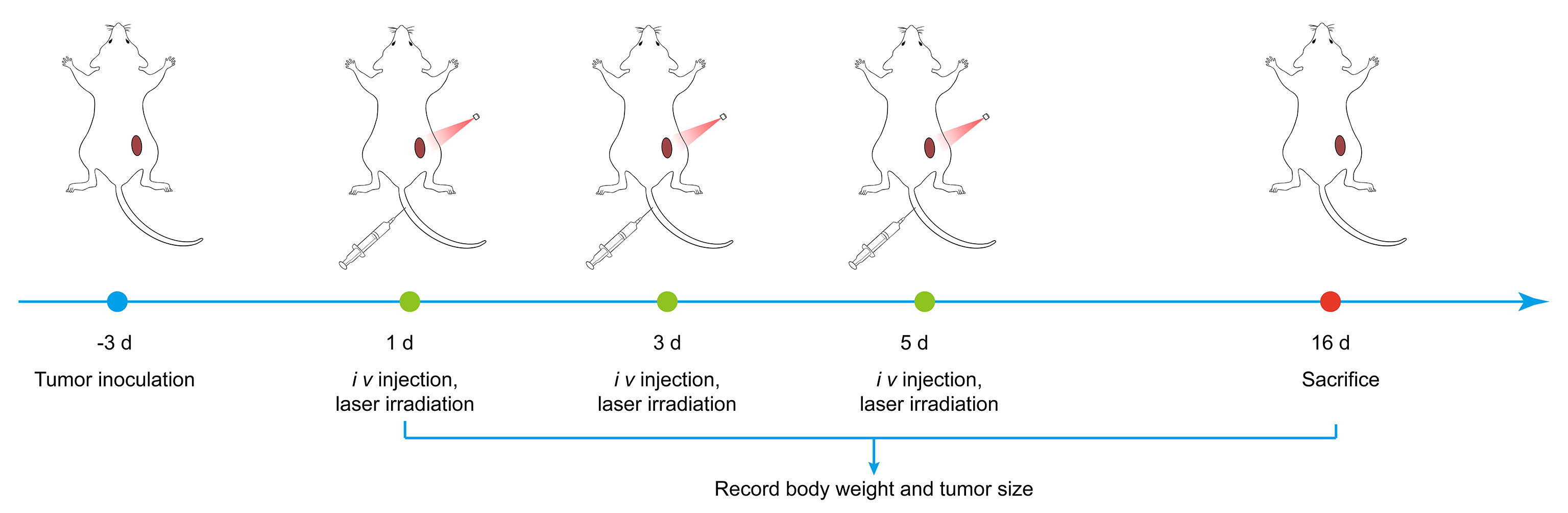


**Fig. S24**. The animal schedule table during the in vivo experiment.

**Fig. S25.** Histological assessments of different organs using H&E staining in the different treatment groups. Scale bar: 200 μm.


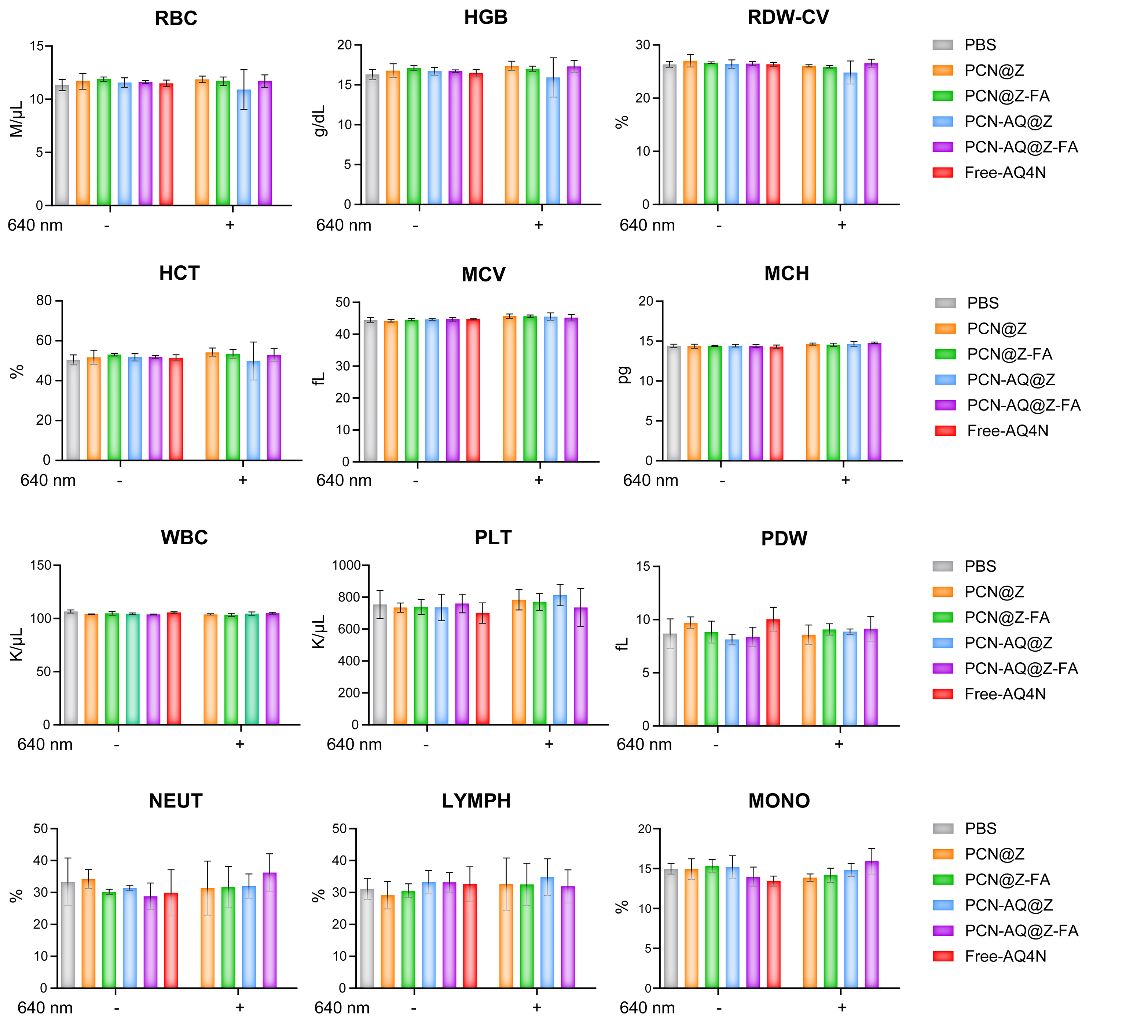


**Fig. S26.** Whole blood analyses from tumor-bearing mice after different treatments. The whole blood indicators include red blood cells (RBC), hemoglobin (HGB), red blood distribution width (RDW), hematocrit (HCT), mean corpuscular volume (MCV), mean corpuscular hemoglobin (MCH), white blood cells (WBC), platelets (PLT), platelet distribution width (PDW), neutrophilic (NEUT), lymphocyte (LYMPH) and monocyte (MONO).


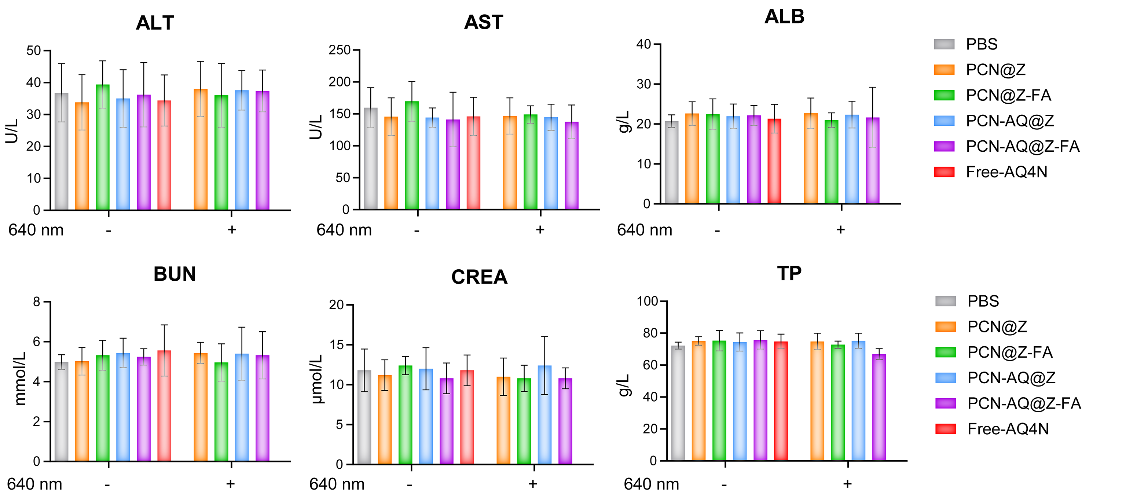


**Fig. S27**. Serum biochemistry analysis of tumor-bearing mice after various treatments to investigate the potential systemic toxicity. The serum biochemical indexes include alanine aminotransferase (ALT), aspartate aminotransferase (AST), albumin (ALB), urea nitrogen (BUN), creatinine (CREA), Total Protein (TP).
